# Life cycle assessment of a commercial rainwater harvesting system compared with a municipal water supply system

**DOI:** 10.1016/j.jclepro.2017.02.025

**Published:** 2017-05-10

**Authors:** Santosh R. Ghimire, John M. Johnston, Wesley W. Ingwersen, Sarah Sojka

**Affiliations:** a ORISE Postdoctoral Research Participant, U.S. Environmental Protection Agency, Office of Research and Development, 960 College Station Rd., Athens, GA 30605, USA; b U.S. Environmental Protection Agency, Office of Research and Development, 960 College Station Rd., Athens, GA 30605, USA; c U.S. Environmental Protection Agency, Office of Research and Development, 26 W. Martin Luther King Dr., Cincinnati, OH 45268, USA; d Randolph College, 2500 Rivermont Ave., Lynchburg, VA 24503, USA

**Keywords:** Life cycle assessment, Commercial rainwater harvesting, Municipal water supply, Energy intensity

## Abstract

Building upon previously published life cycle assessment (LCA) methodologies, we conducted an LCA of a commercial rainwater harvesting (RWH) system and compared it to a municipal water supply (MWS) system adapted to Washington, D.C. Eleven life cycle impact assessment (LCIA) indicators were assessed, with a functional unit of 1 m^3^ of rainwater and municipal water delivery system for toilets and urinals in a four-story commercial building with 1000 employees. Our assessment shows that the benchmark commercial RWH system outperforms the MWS system in all categories except Ozone Depletion. Sensitivity and performance analyses revealed pump and pumping energy to be key components for most categories, which further guides LCIA tradeoff analysis with respect to energy intensities. Tradeoff analysis revealed that commercial RWH performed better than MWS in Ozone Depletion if RWH’s energy intensity was less than that of MWS by at least 0.86 kWh/m^3^ (249% of the benchmark MWS energy usage at 0.35 kWh/m^3^). RWH also outperformed MWS in Metal Depletion and Freshwater Withdrawal, regardless of energy intensities, up to 5.51 kWh/m^3^. An auxiliary commercial RWH system with 50% MWS reduced Ozone Depletion by 19% but showed an increase in all other impacts, which were still lower than benchmark MWS system impacts. Current models are transferrable to commercial RWH installations at other locations.

## 1. Introduction

Approximately 5–20% of the global population is predicted to live under absolute water scarcity (<500 m^3^/person/year) up until the point of a 2 °C increase in mean global temperature, and a higher percentage as temperatures rise further (Schewe et al., 2014). The climate change impact and the 21st century’s megadroughts with longer duration may result in unprecedented water scarcity from the southwestern to the southeastern U.S., and to other parts of the world, which is driving the global search for alternate water sources (USGCRP, 2012; Ault et al., 2016; USEPA, 2016a). Rainwater harvesting (RWH) is receiving renewed interest as a green infrastructure practice in the face of necessary adaptation to global climate change (USGCRP, 2012; Ghimire et al., 2014). RWH has been used for drinking water and agricultural irrigation since 11,500 BCE in North America and 4500 BCE in Asia (Pandey et al., 2003; Mays et al., 2013; Ghimire and Johnston, 2015). Other potential benefits of RWH include reduced impacts on the environment and human health, reduced stormwater runoff and combined sewer overflows, and economic viability (Ghimire et al., 2012; Wang and Zimmerman, 2015). In practice the implementation of RWH has remained a challenge. This is due primarily to a lack of understanding of its environmental and human health impacts (criteria pollutants from material selection and energy use) and partly due to the lack of regulations governing RWH practices. Although state regulations and statutes continue to favor RWH (ARCSA, 2016; Harvesth2o, 2016; NCSL, 2016), the U.S. Environmental Protection Agency (EPA) reported “there are currently no federal regulations governing rainwater harvesting for non-potable use, and the policies and regulations enacted at the state and local levels vary widely from one location to another” (USEPA, 2013a).

Studies worldwide have explored RWH life cycle cost impacts (Roebuck et al., 2011), water savings potential (Ghisi et al., 2009; Domenech and Sauri, 2011; Ward et al., 2012), water quality and health risks (Lye, 2002; Domenech et al., 2012), optimal designs (Sample and Liu, 2014), energy intensity versus economic and CO_2_ emissions (Gurung and Sharma, 2014; Siems and Sahin, 2016), hydrologic impacts (Ghimire and Johnston, 2013), and climate change adaptation (Pandey et al., 2003). Of particular interest is the life cycle environmental and human health criteria air pollutant impacts of RWH. Life cycle assessment (LCA) has been widely used since its inception in the late 1960s in diverse sectors to assess environmental and human health impacts in a cradle-to-grave approach that avoids or minimizes unintended consequences (Hunt et al., 1996; ISO, 2006b, a; Meyer and Upadhyayula, 2013; Ingwersen et al., 2016). Use of LCA to assess RWH systems is also increasing. Previous studies focused on residential small and large building systems that vary by region; however, findings varied by design parameters and data sources, as demonstrated by the following examples. Crettaz et al. (1999) performed an LCA on RWH for clothes washing and toilet flushing in Switzerland and reported RWH as energetically favorable if pumping energy intensity for municipal drinking water was greater than 0.8 kWh/m^3^. Angrill et al. (2012) reported life cycle impacts of RWH for laundry use in compact urban densities were generally lower than in diffused settings in Spain. Morales-Pinzón et al. (2015) reported a lower life cycle global warming potential of RWH than tap water for toilet flushing and clothes washing in a single-family house if the average storage tank was smaller than 2 m^3^.

In the U.S., interest in RWH is growing due to increased droughts as well as its environmental benefits (Thomas et al., 2014; Sojka et al., 2016). An energy and greenhouse gas (GHG) emission analysis of RWH in a university building in Ohio reported RWH as a viable option with GHG emission payback periods higher than the energy payback periods but contradicted the results of Anand and Apul (2011) due to difference in data sources (process-based versus Economic Input Output life cycle assessment data) (Devkota et al., 2015). Wang and Zimmerman (2015) assessed life cycle climate change, fossil fuel depletion, and eutrophication impacts of RWH for office buildings in 14 large cities across the U.S., from Boston, Massachusetts to Seattle, Washington, and reported that reduced eutrophication and combined sewer flows varied with location.

Ghimire et al. (2014) published an LCA of domestic and agricultural RWH systems that compared conventional water supplies, municipal water supplies and well water irrigation systems in the southeastern U.S. The American Rainwater Catchment Systems Association then contacted the authors about conducting a similar study for a commercial RWH system. Comprehensive understanding of the life cycle implications of commercial RWH systems is only in its infancy but is important for informing urban water management planning and decision making. Consequently, we conducted an LCA of a typical commercial RWH system in an average size commercial building sited in a large U.S. city (EIA, 2016) and combined this with scenario and sensitivity analysis, with the intention of providing more generalizable life cycle implications of commercial RWH systems.

### 1.1. Objective, scope, and novelty

Our objective is to conduct an LCA of a commercial RWH system and compare it to a municipal water supply (MWS), hereafter called benchmark commercial RWH and MWS systems. Eleven life cycle impact assessment (LCIA) indicators were calculated per functional unit of 1 m^3^ of rainwater and municipal water delivery for flushing toilets and urinals in a four-story commercial building with 1000 employees. These included Acidification, Energy Demand, Eutrophication, Fossil Depletion, Freshwater Withdrawal, Global Warming, Human Health Criteria, Metal Depletion, Ozone Depletion, Smog, and Evaporative Water Consumption. LCIA sensitivity was addressed for (i) storage tank materials and volume, (ii) energy usage or energy intensity, (iii) water demand, (iv) water loss, (v) system service life, and (vi) an auxiliary commercial RWH system augmented with MWS. The LCA system boundary spans cradle-to-grave, excluding the distribution of both systems’ components from final manufacture to point of use and disposal phases for lack of comparable data (see Supplementary Material or SM 1 for additional details). This study provides a comprehensive LCA of commercial RWH to inform RWH planning and decision making, with standards and regulations at state and local levels governing urban water management decisions (USEPA, 2013a). The life cycle inventory (LCI) and LCA models are generally transferrable in recreating LCA models of other commercial RWH and MWS systems by obtaining appropriate LCI data. The following sections describe methods, tools, databases and assumptions and results, with concluding remarks on potential implications for commercial RWH design and planning.

## 2. Methods and tools

### 2.1. Site selection

Washington, D.C. was selected as the study site primarily due to readily available data on the MWS system, precipitation record and commercial RWH design. While the commercial RWH system was designed for local precipitation patterns, the analysis required extensive data on an existing urban MWS system compatible with existing life cycle data for MWS. The EPA conducted a study of the Cincinnati MWS system that uses the Ohio River as a source with chlorine disinfection (Cashman et al., 2014). Data were developed in a modular and reusable fashion to be reconfigured and customized for other regions. Study area selection criteria included a large urban area with similar source water characteristics, treatment processes and distribution architecture to Cincinnati, Ohio, as well as publically available data on the water supply system and sufficient precipitation record. Other U.S. cities considered included Los Angeles, Phoenix, Austin, Chicago and Atlanta. Although water demand for Washington, D.C. (benchmark MWS system) was slightly higher ≈ 1.1 times Cincinnati MWS system demand, it has comparable treatment system and distribution pipe network composition (Ductile Iron pipe >90%).

### 2.2. Definition of the benchmark commercial RWH and MWS systems

**Fig. 1.**
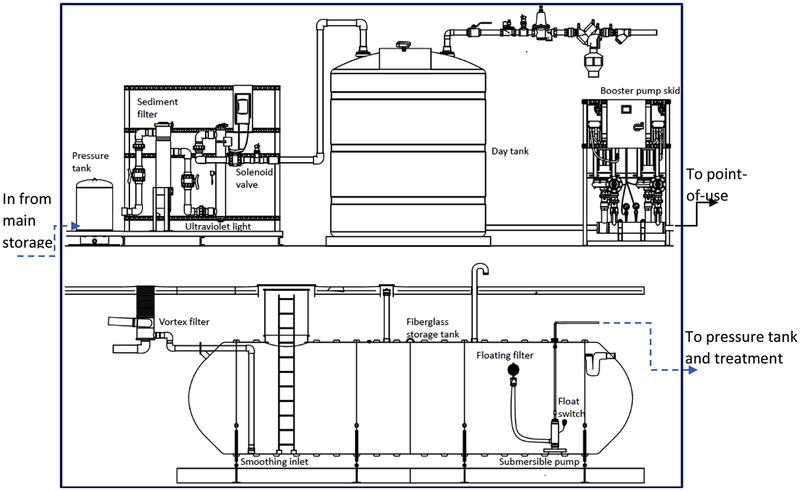
Schematic of commercial rainwater harvesting system (designed by Rainwater Management Solutions).

The American Rainwater Catchment Systems Association provided the design for a commercial RWH system from one of its member companies to be configured for a typical urban system. This design was customized for flushing 40 toilets and 15 urinals in a four-story commercial building with 1000 people, adapted to Washington, D.C. (Fig. 1 and Table 1, see SM 1 and 2 for additional details).

Key design parameters and assumptions used in the benchmark commercial RWH system analysis were:

- Total water demand forflushing all urinals and toilets for the building was estimated at 2653 m^3^/y (2685 gallons/day): high-efficiency urinal demand at 0.47 L per flush (l/f) or 0.125 gallon/flush (g/f), and high-efficiency toilet demand at 4.8 l/f or 1.28 g/f (AWE, 2016);
- Storage tank volume was designed at 76 m^3^ (20,000 gallons), supplying 77% of total volumetric water demand (RMS, 2009) (see SM 1 for additional details);
- The benchmark commercial RWH system met 77% of total toilet and urinal water demand (77% of 2653 m^3^/y = 2042.81 m^3^/y) and an auxiliary commercial RWH system was operated with support of MWS to meet additional demand;
- Pumping energy intensity was estimated at 0.19 kWh/m^3^ (see SM 1 for additional details);
- Water loss throughout the system was 5%;
- Service life of the system was 50 years and components with shorter service lives were replaced at the end of their service lives;
- Distribution of system components from final manufacture to point of use and disposal were excluded from LCA.

**Fig. 2.**
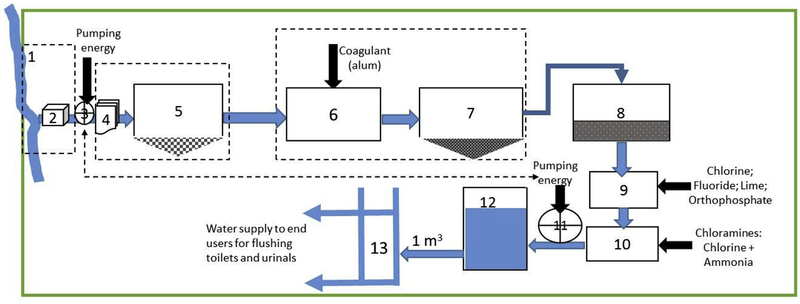
Washington, D.C. municipal water supply system. Number Key: (1) Source water (Potomac River); (2) Source water acquisition infrastructure; (3) Acquisition pump; (4) Screening infrastructure; (5) Pre-sedimentation (natural settling in a pre-treatment storage); (6) Flocculation; (7) Sedimentation (coagulated particles); (8) Filtration (sand filter); (9) Primary Disinfection (Gaseous Chlorine, Lime addition, Fluorination, Orthophosphate); (10) Secondary Disinfection: Chloramines; (11) Distribution Pump (four); (12) Storage Tanks (eleven); (13) Water pipe network (based on District of Columbia Water and Sewer Authority (DCWSA, 2015) and Baltimore District, U.S. Army Corps of Engineers (USACE, 2016)).

The benchmark MWS system was defined using available information from the District of Columbia Water and Sewer Authority (DCWSA, 2015) and Baltimore District, U.S. Army Corps of Engineers (USACE, 2016) (Fig. 2 and SM 2).

The District of Columbia Water and Sewer Authority (DC Water) purchased 100% of drinking water from the Washington Aqueduct operated by the Baltimore District, U.S. Army Corps of Engineers. The Washington Aqueduct collected, purified and pumped drinking water to three jurisdictions, including DC Water, Arlington County, Virginia, and Fairfax County Water Authority, Virginia (USACE, 2016). Washington Aqueduct owned and operated two treatment plants, Dalecarlia and McMillan (source water: Potomac River), an intake pumping facility, and three water storage facilities. Seventy-two percent of total treated water from the two plants (i.e., 72% × 192,052,776 m^3^/y) was sold to DC Water in 2012. DC Water consisted of 2092 km (1300 miles) of pipes, eight water storage facilities, four pumping stations, and 36,000 valves. Thus, DC Water (benchmark MWS system) was a combination of District of Columbia Water and Sewer Authority-owned and Washington Aqueduct-owned facilities. Key design parameters and assumptions pertaining to the benchmark MWS system were:

- The two water treatment plants of Washington Aqueduct produced 192,052,776 m^3^/y or 139 million gallons of water per day and 72% of the treated water from the Aqueduct sold to DC Water;
- Water loss throughout the system was 24%, based on sold to pumped ratio of 76% in 2008 (DCWSA, 2008);
- Water treatment options were consistent with average U.S. water treatment;
- MWS energy intensity was estimated at 0.35 kWh/m^3^ combining two energy uses: Washington Aqueduct energy use (0.20 kWh/m^3^) included electricity required to pump source water and energy throughout the water treatment plants and DC Water services pumping energy use (0.15 kWh/m^3^) for transporting the treated water (see SM 1 for additional details);
- All infrastructure had service lives of 100 years except the pump with 15-y service life, replaced at the end of its service life;
- Distribution of the system components from final manufacture to point of use and disposal were excluded from LCA.

#### 2.2.1. Functional unit

To facilitate comparison of water supply from two different sources, 1 m^3^ of water supply was defined as the functional unit for annual water supply and service lives of system components. Standardization by 1 m^3^ enabled comparison of LCIA scores regardless of volumetric water supply, because material input is linearly related to volumetric water supply. In addition, accounting for service lives of system components addressed the issue of product replacement.

### 2.3. Development of LCI and LCA models

Primary data collected on commercial RWH included design components (Table 1, also see SM 2). Similarly, primary data were collected on the benchmark MWS system for system components, amount of material and energy use, and water demand from the District of Columbia Water and Sewer Authority (DCWSA, 2015) and Baltimore District, U.S. Army Corps of Engineers (USACE, 2016) (see SM 2 for details on LCI). The LCI of the benchmark systems was compiled using Building for Environmental and Economic Sustainability (BEES) (NIST, 2013), Ecoinvent (2012) version 2.2, U.S. LCI database (NREL, 2013), and Cincinnati drinking water treatment and distribution systems collected from a previous study (Cashman et al., 2014). There are limitations to using the Ecoinvent database, a European LCI, in the U.S. because results would be sensitive to data quality and material type. However, data availability is a major consideration, such that the LCI and LCA utilized data and methods from a domestic RWH study in the southeastern U.S (Ghimire et al., 2014). as well as the LCI of the Cincinnati MWS system (Cashman et al., 2014).

### 2.4. Life cycle impact assessment (LCIA)

LCIA methods included combining the Tool for the Reduction and Assessment of Chemical and Other Environmental Impacts or, TRACI 2.1 (USEPA, 2013b), with the ReCiPe’s Metal Depletion and Fossil Depletion (Goedkoop et al., 2012), Water Footprint’s Evaporative Water Consumption and Freshwater Withdrawal (WFN, 2009), and the non-renewable Cumulative Energy Demand of Ecoinvent (Hischier et al., 2010). TRACI 2.1 methods included were Global Warming, Ozone Depletion, Smog, Human Health Criteria (air pollutants), Eutrophication, and Acidification. Human health (cancer) and ecotoxicity indicators were excluded due to lack of high quality life cycle data for release to air and water, consistent with Ingwersen et al. (2016). OpenLCA (2016) version 1.4.2, an open source LCA software (GreenDelta ^©^ 2016) was used for all LCIA calculations in conjunction with LCIA methods and LCI databases.

LCIA percentage contributions of the benchmark systems and their components were analyzed. LCIA sensitivity analysis was conducted for various scenarios (Table 2) which further guided LCIA tradeoff analysis with respect to energy intensities and the auxiliary commercial RWH system. Sensitivity and tradeoff LCIA results were normalized for each impact category by maximum LCIA score (see SM 4 for the details on LCIA normalization).

LCIA tradeoff of the auxiliary commercial RWH system, augmented with 10%–90% MWS, was assessed by summing the fractional impacts of both systems delivering 1 m^3^ of water supply (Equation (1)):

(1) $$I_{aux}=p_{c}\times I_{c}+p_{m}\times I_{m}$$

for each impact category*I*_*aux*_ = the auxiliary system’s impact (impact/m^3^ of water supply), .*I*_*c*_ = the benchmark commercial RWH system’s impact (impact/m^3^ of water supply), .*I*_*m*_ = the benchmark MWS system’s impact (impact/m^3^ of water supply), .*p*_*c*_ and *p*_*m*_ are commercial RWH and MWS percentage (in decimal) such that

(2) $$p_{c}+p_{m}=1$$

Equation (1) was applied to estimate the impacts of the auxiliary commercial RWH system using the functional unit impacts of both benchmark systems; e.g., if a commercial RWH system supplied 70% of total demand (i.e., *p*_*c*_ = *0.7*), then the value of *I*_*aux*_ would be 0.7 × benchmark commercial RWH system impact, *I*_*c*_, + 0.3 × municipal water supply impact, *I*_*m*_. Functional unit impacts per 1 m^3^ water delivery were calculated, with inputs linearly related to volumetric water supply.

## 3. Results and discussion

### 3.1. Performance analysis

**Fig. 3.**
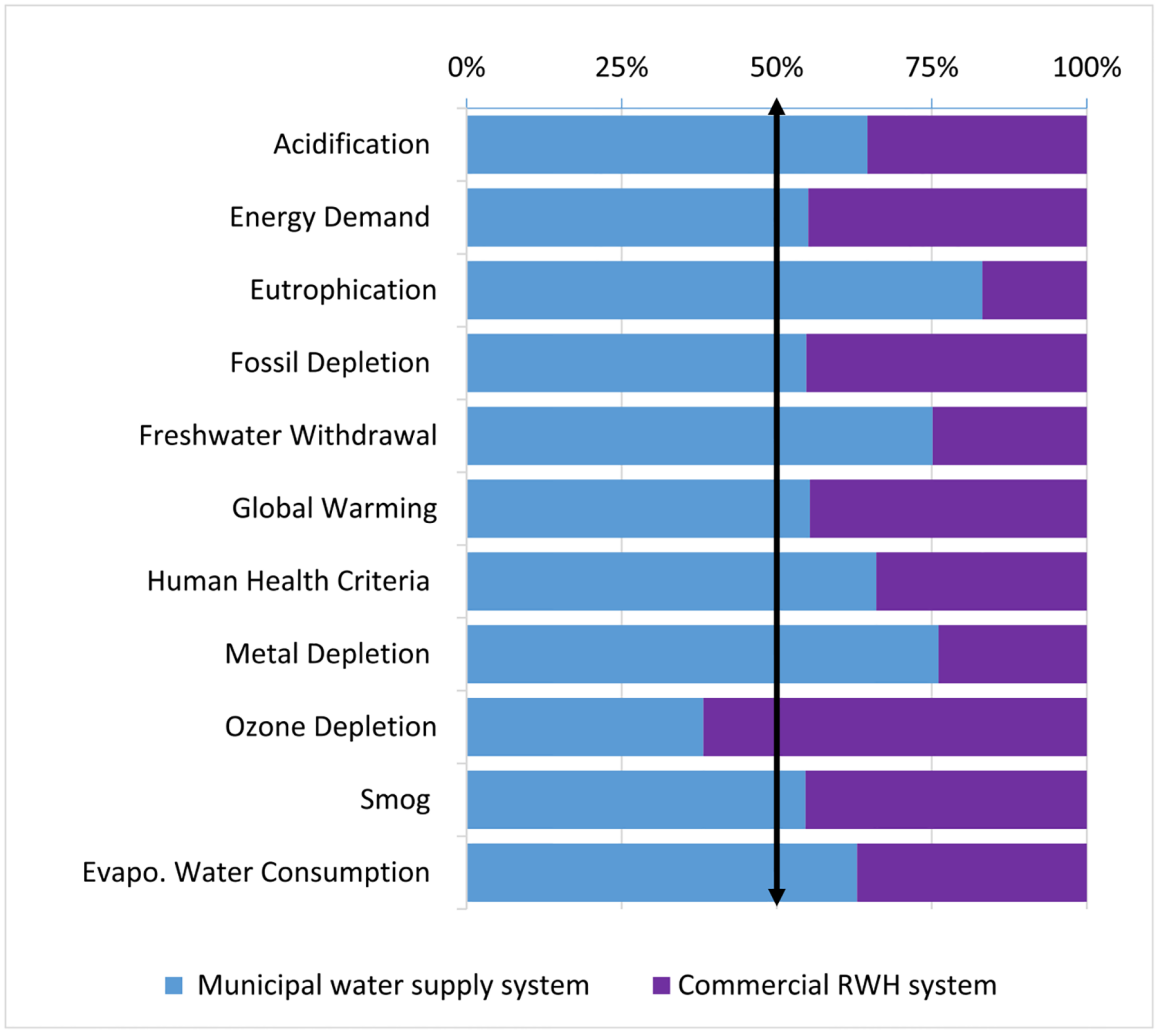
Comparison of life cycle impacts of the benchmark commercial rainwater harvesting system to the municipal water supply system adapted to Washington, D.C. (vertical arrow indicates the threshold 50%).

LCIA percentage contribution analysis showed that the benchmark commercial RWH system performed better than (<40%) or equivalent to (45%–55%) the MWS system in all impact categories except Ozone Depletion at 62% (Fig. 3).

**Fig. 4.**
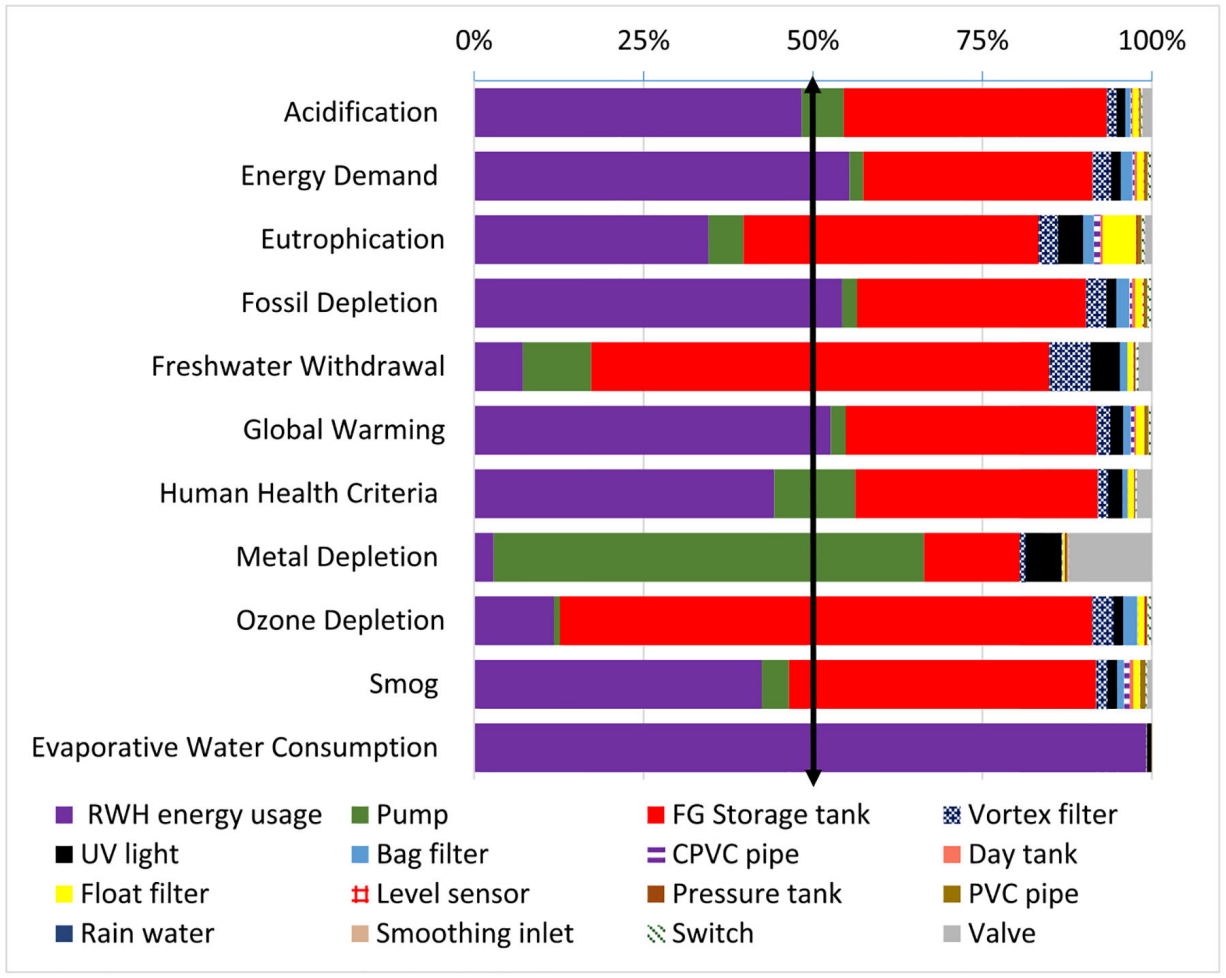
Percentage comparison of life cycle impacts showing the performance of commercial rainwater harvesting (RWH) system components. Note: RWH energy usage includes pumping energy at the commercial RWH storage tank (vertical arrow indicates the threshold 50%).

**Fig. 5.**
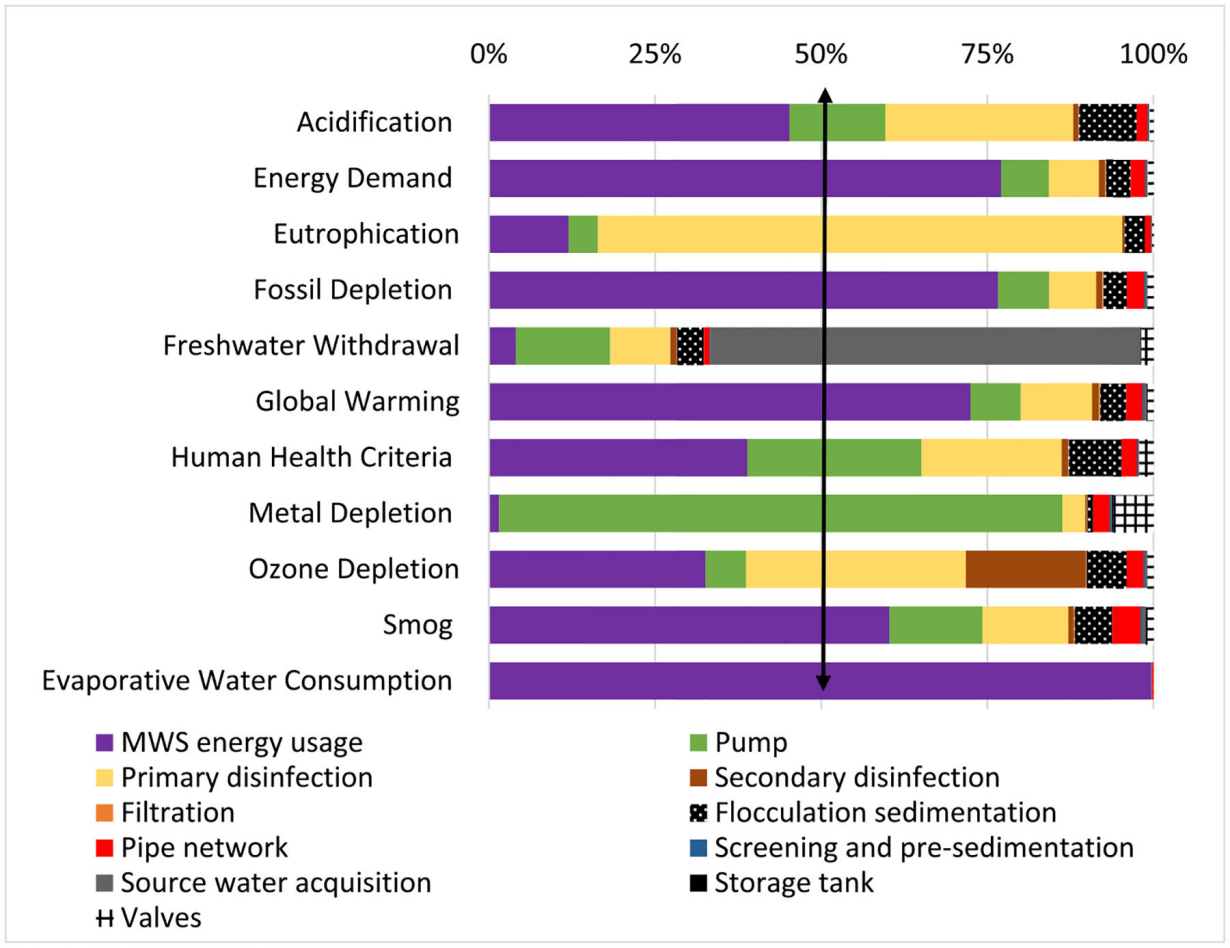
Percentage comparison of life cycle impacts showing the performance of municipal water supply (MWS) system components. Note: MWS energy usage includes electricity required to pump in source water, energy throughout the water treatment plants, and pumping energy for transporting treated water (vertical arrow indicates the threshold 50%).

Commercial RWH was slightly better than or equivalent to (at 45%, assuming a margin of error) MWS for Energy Demand, Fossil Depletion, Global warming, and Smog and better than MWS in 6 impact categories, ranging from 65% (Acidification) to 83% (Eutrophication). Percentage comparison of component-specific LCIA revealed energy usage to be the dominant contributor of both systems (Figs. 4–5). Commercial RWH energy usage (pumping energy) and storage tank collectively dominated (>50%) in all but Metal Depletion, ranging from 74.8% (Freshwater Withdrawal), 89.6% (Global Warming), to 99.2% (Evaporative Water Consumption). The commercial RWH storage tank (Fiberglass) dominated in Ozone Depletion (78.5%) and Freshwater Withdrawal (67.6%). MWS energy usage (treatment plant operation and water transport) dominated (>50%) in five categories, ranging from 60.3% (Smog) to 99.7% (Evaporative Water Consumption). The pump dominated in Metal Depletion impact of both systems, 63.5% in commercial RWH and 84.8% in MWS. Source water acquisition contributed to total MWS Freshwater Withdrawal impact at 64.8%, and primary and secondary disinfection together contributed to Eutrophication and Ozone Depletion impacts at 79.2% and 51.1%. All other commercial RWH system components were below 15%, and the remaining MWS system components were <10% (Figs. 4–5). Detailed values of LCIA of benchmark systems and their components are included in SM 2.

### 3.2. Sensitivity analysis

**Fig. 6.**
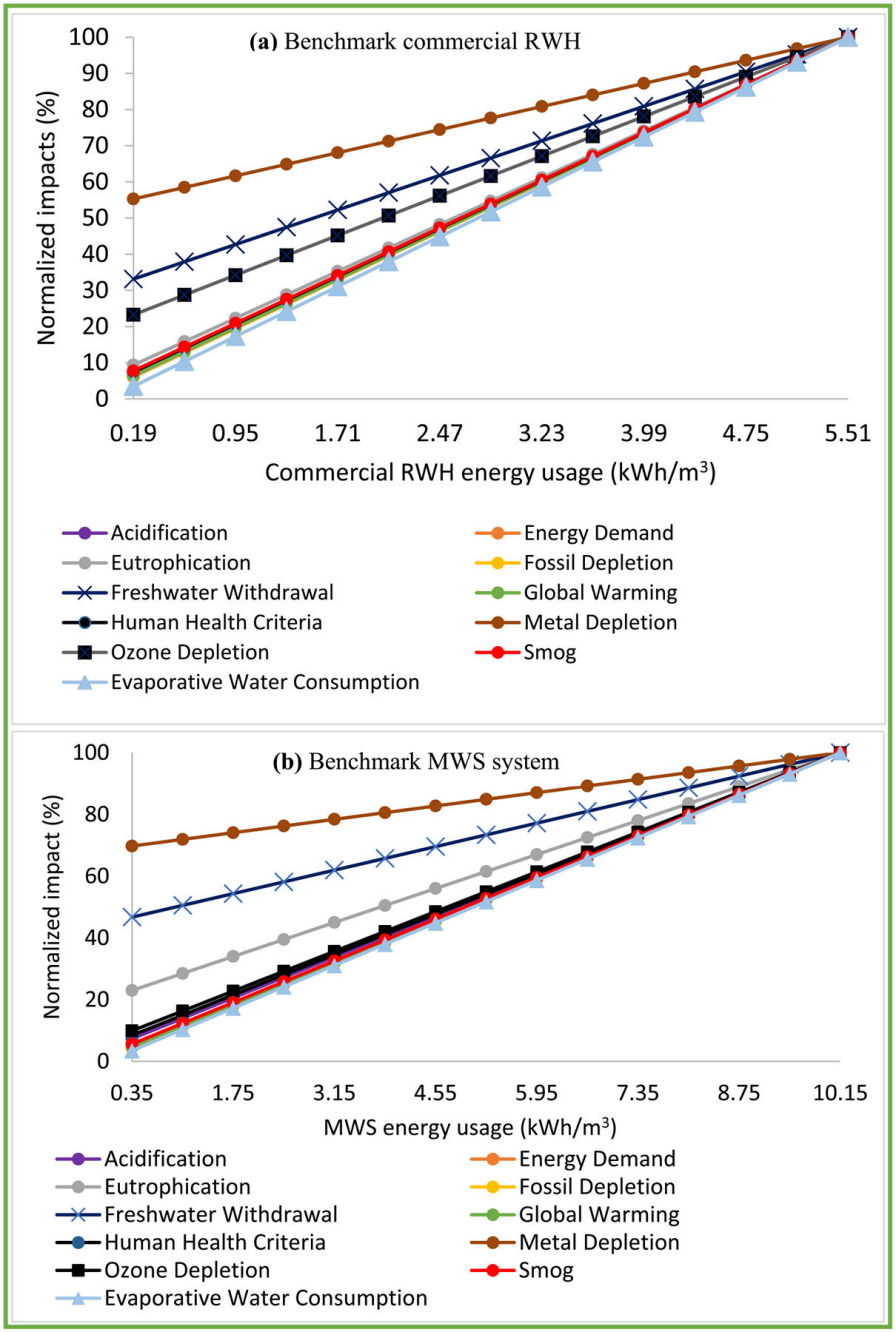
Sensitivity analysis of energy usage to life cycle impacts of (a) benchmark commercial rainwater harvesting (RWH) system and (b) benchmark municipal water supply (MWS) system.

Normalized LCIA of energy usage revealed linear impact variation, confirming linearity in LCIA scores consistent with linear LCA models (Fig. 6). For purposes of this analysis, impact variation were grouped as Mild slope, e.g., Metal Depletion; Moderate slope, e.g., Freshwater Withdrawal; or High slope, e.g., Evaporative Water Consumption (Fig. 6a–b).

The rate of change of impacts across LCIA categories was consistent with the dominant components. For example, in the case of commercial RWH energy usage, Evaporative Water Consumption and Fossil Depletion fell under High slope because pumping energy dominated those impacts; Ozone Depletion and Freshwater Withdrawal fell under Moderate slope because of storage tank. LCIA scores, normalized with respect to maximum scores of the storage tank, showed that fiberglass material dominated nine impact categories, while polyethylene (PE) tank dominated Energy Demand and Fossil Depletion (see SM 4). The fiberglass storage tank material was deemed appropriate for underground placement as designed by Rainwater Management Solutions. In practice, tank material is selected to meet system requirements and impact focus. Linear slopes were also observed in commercial RWH storage tank volume, water demand, water loss, and auxiliary commercial RWH system sensitivity analyses (SM 4). Additional sensitivity analysis results of storage tank materials and volume, water demand, water loss, system service life and auxiliary commercial RWH system, as well as a closer look at the LCIA release contributing processes of commercial RWH storage tank, pumping energy and pump, are provided in SM 3–4.

### 3.3. The LCIA tradeoffs of the systems

**Fig. 7.**
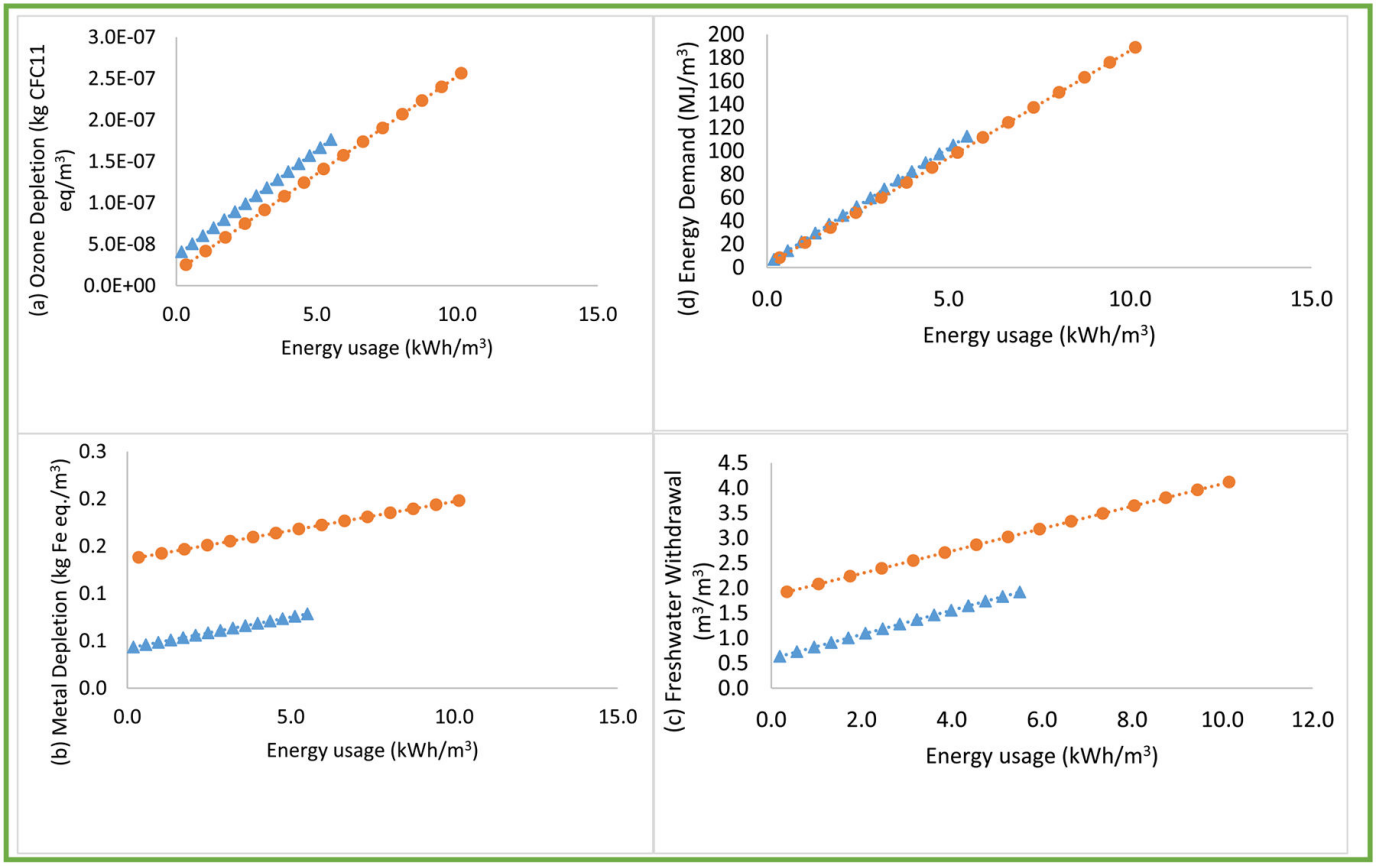

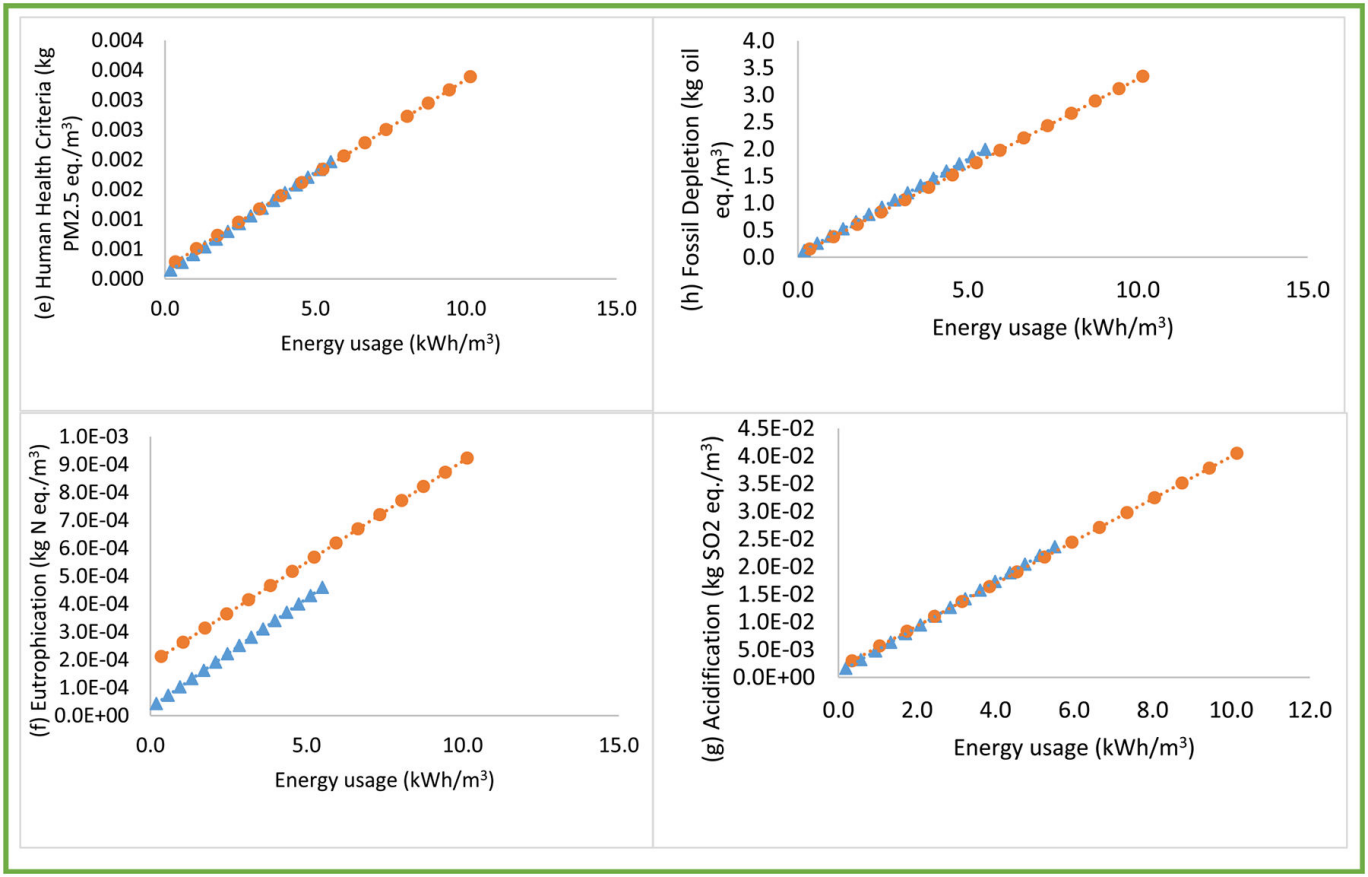

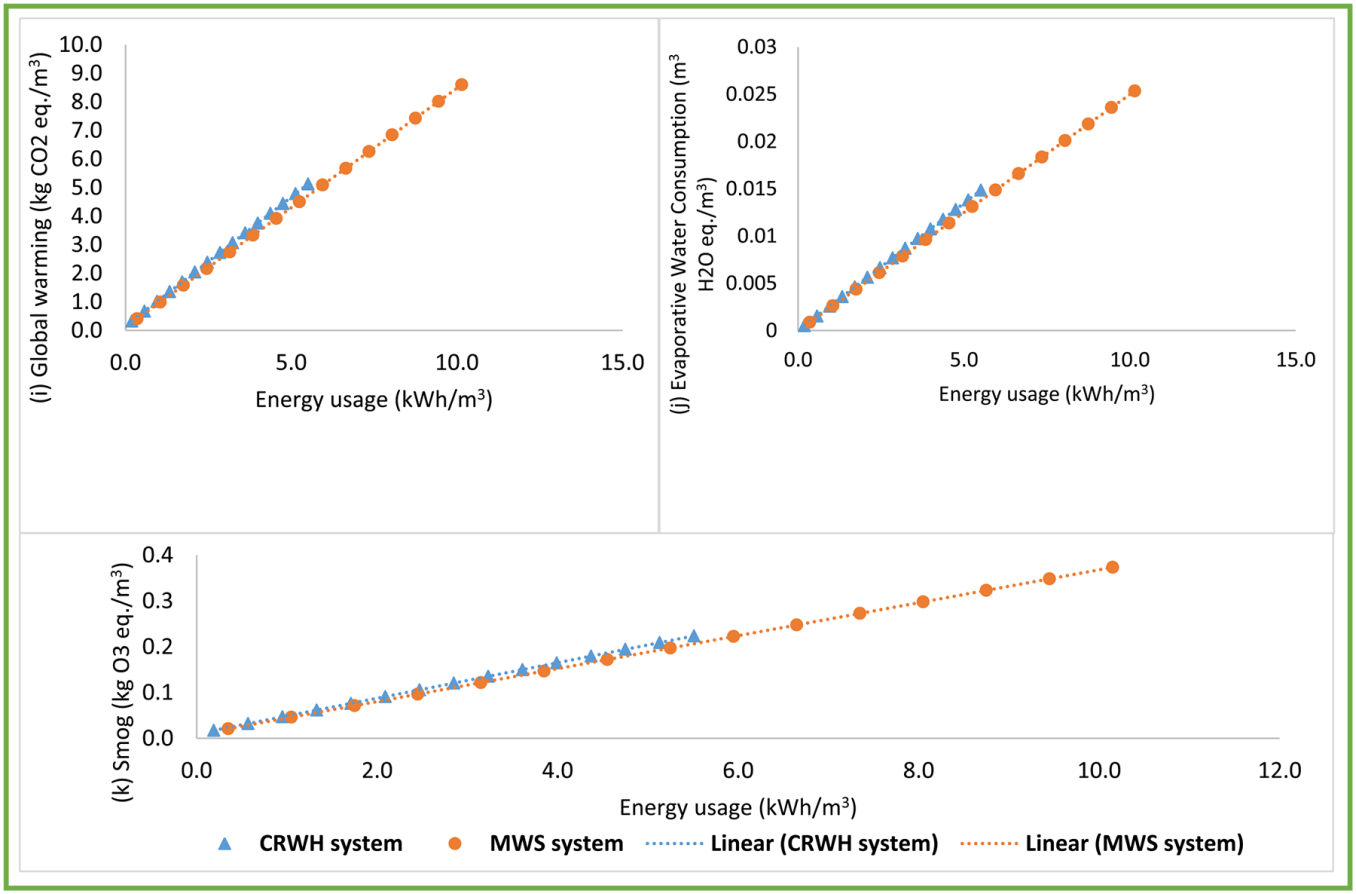
LCIA tradeoff analysis for a range of energy intensities in commercial rainwater harvesting (RWH) and municipal water supply (MWS) systems: (a) Ozone Depletion, (b) Metal Depletion, (c) Freshwater Withdrawal, and (d) Energy Demand categories. Legend: Triangles = commercial RWH system and Circles = MWS system, (e) Human Health Criteria, (f) Eutrophication, (g) Acidification, and (h) Fossil Depletion. Legend: Triangles = commercial RWH system and Circles = MWS system, (i) Global warming, (j) Evaporative water consumption and (k) Smog.

Energy usage or energy intensity sensitivity analysis revealed conditional LCIA tradeoffs of commercial RWH and MWS systems to energy requirements (Fig. 7a–k). Using regression equations (Fig. 7, Table 3), LCIA tradeoffs can be predicted for the two systems when energy intensity is known.

For Ozone Depletion, commercial RWH outperformed MWS if commercial RWH pumping energy intensity *(E*_*c*_*)* was less than MWS energy intensity (*E*_*m*_), *E*_*c*_ *+* 0.86 ≤ *E*_*m*_. For example, for *E*_*c*_ = 0.19 kWh/m^3^ and *E*_*m*_ = 1.05 kWh/m^3^, Ozone Depletion scores of commercial RWH and MWS systems were estimated at 4.1 × 10^−8^ kgCFC11 eq/m^3^ and 4.2 × 10^−8^ kgCFC11 eq/m^3^. Commercial RWH outperformed in Metal Depletion and Freshwater Withdrawal impacts, regardless of energy intensities analyzed. In Human Health Criteria and Eutrophication impacts, commercial RWH outperformed MWS, depending on energy intensities, if *E*_*c*_ ≤ *E*_*m*_. Commercial RWH outperformed in all other impact categories such as Energy Demand, if *E*_*c*_ + 0.09 ≤ *E*_*m*_. Mean Absolute Percentage Error (MAPE) of predicting LCIA impact scores using commercial RWH Tradeoff equations ranged from 0.26% (Evaporative Water Consumption) to 1.20% (Ozone Depletion) (Table 3; also see SM 4 for additional details on MAPE). In the case of MWS Tradeoff equations, MAPE ranged from 0.12% (Smog) to 2.40% (Metal Depletion). MAPE was selected as an appropriate, transparent statistic for comparing fit of the equations instead of another statistic such as coefficient of determination (R^2^), thus avoiding any potential model exaggeration as reported by Birnbaum (1973).

Auxiliary commercial RWH system sensitivity analysis provided additional insights into LCIA tradeoffs. The impacts of the auxiliary system increased linearly with the percentage increase of MWS, as explained by Equation (1), except for Ozone Depletion with the reverse relationship due to the greater Ozone Depletion impact of benchmark commercial RWH system than MWS. An auxiliary commercial RWH system with 50% MWS reduced Ozone Depletion by 19%, but with an increase in all other impacts ranging from 10% Smog, 11% Energy Demand, 35% Evaporative Water Consumption, to 197% Eutrophication with respect to the benchmark commercial RWH system (Table 4). All increases were below benchmark MWS system impacts though, by as much as 8% Smog to 40% Eutrophication.

The benchmark commercial RWH storage tank volume of 76 m^3^ (20,000 gallons) was estimated using a traditional behavioral and mass balance model, a spreadsheet-based, time series modeling approach (RMS, 2009). Two tank materials were evaluated by LCIA score, PE versus fiberglass, utilizing BEES and Ecoinvent data sources. Storage tank material and volume depends on the system requirement, annual precipitation and impact focus. In addition, infrastructure characteristics such as pump efficiencies, number of stories in a building and system head, as well as geographic characteristics such as location and water demand, water sources, treatment processes and storage options influence pumping energy intensities. Variations with sensitivity analyses of commercial RWH energy intensities (0.19 kWh/m^3^ to a hypothetical value of 5.51 kWh/m^3^) and MWS energy intensities (0.35 kWh/m^3^ to a hypothetical value of 10.15 kWh/m^3^) were addressed by capturing theoretical and empirical commercial RWH pumping energy (0.20 kWh/m^3^ to 4.9 kWh/m^3^) (Retamal et al., 2009; Vieira et al., 2014) and average national MWS pumping energy estimates (0.396 kWh/m^3^ to high intensity water source desalination of 3.17 kWh/m^3^) (Pabi et al., 2013; Wang and Zimmerman, 2015). Although alternative disinfection options were not the focus of current study, it is important to note that primary and secondary disinfection dominated Eutrophication and Ozone Depletion impacts of the MWS system. Therefore, appropriate information on disinfection technology is important in comparing impacts with a commercial RWH system.

The benchmark MWS system used chlorination similar to a majority of U.S. water systems—eighty-five percent of the water treatment plants in the U.S. were using chlorine by 1941, and today all drinking water filtration in the U.S. is accompanied by chlorination or other disinfection technologies such as ozonation and UV light (Sedlak, 2014).

## 4. Summary and study implications

In addition to assessing the comprehensive life cycle environmental and human health impacts of a commercial RWH system compared to a MWS system, we addressed sensitivity of LCIA impact scores to storage tank material and volume, energy usage, water demand, water loss, system service life and an auxiliary commercial RWH system augmented with MWS. The LCA system boundary spans cradle-to-grave, excluding distribution of both systems’ components from final manufacture to point of use and disposal phases.

Our analyses revealed that the benchmark commercial RWH performed better or equivalent (45–55%) to MWS in all impact categories except Ozone Depletion. Sensitivity analyses of energy usage, water demand, water loss, and storage tank volume confirmed linearity trends in LCIA scores. Additional sensitivity analyses showed that storage tank with fiberglass material dominated in nine of the 11 impact categories, except in Energy Demand and Fossil Depletion. Annual LCIA impacts for the commercial RWH system with longer service life (75 y) were lower than that of the system with shorter service life (50 y). An auxiliary commercial RWH system augmented with 50% MWS reduced Ozone Depletion impact by 19% showing increases in all other impacts. The LCIA tradeoff equations with respect to energy usage revealed conditional LCIA tradeoffs with energy requirements.

This study informs RWH planning and decision making through a comprehensive LCA of a commercial RWH system, with standards and regulations at the state and local level (USEPA, 2013a). LCA models are transferrable to other commercial RWH installations and study implications are summarized:

- A suite of 11 LCIA impact categories, as well as sensitivity analyses, provided insights into LCIA impact tradeoffs. The importance of LCIA impact categories and dominant components, especially material selection and energy usage, should be considered.
- Commercial RWH fiberglass storage tank material dominated (>50%) in Ozone Depletion and Freshwater Withdrawal impact categories. Selecting PE instead of fiberglass to reduce Ozone Depletion impact may be an option, although a PE tank may not be as appropriate for underground placement because it also necessitates greater Energy Demand and Fossil Depletion than fiberglass. Sensitivity of the data source was evaluated by performing an example LCIA of commercial RWH storage tank materials, fiberglass and PE with Ecoinvent (2012) and the BEES database (NIST, 2013). A data quality assessment of LCI by defining representativeness (including temporal, geographic, technological aspects, and completeness of data) would provide insight into variation in data (ISO, 2006b; USEPA, 2016b); however, it was beyond the scope of this study.
- Commercial RWH pumping energy usage was the most dominant component; therefore, eliminating or reducing pumping energy is key to reducing commercial RWH impacts. An alternate energy mix (e.g., solar) can minimize impacts of a dominant release contributor.
- Regression equations are useful for estimating impact tradeoffs of alternatives. Benchmark MWS treatment practices were consistent with typical U.S. surface water treatment, thus applicable to other MWS systems that use surface water such as New York City (York, 2016), Austin (Water, 2010), and Seattle (2016). Accurate information on water treatment and energy use is imperative, and caution must be exercised when estimating the impacts of an energy-efficient MWS system because these systems require significantly less energy than energy-intensive ones (e.g., water source desalination and water transportation).
- LCIA tradeoffs exist due to variations in system requirements such as energy intensities and tank size for different rainfall and water demands. While component performance findings can help to improve the design of a specific component, slopes and tradeoff results focus on target impacts of a commercial RWH system.
- Appropriate storage tank volume, water demand, and energy intensity may be determined by considering a threshold point to minimize commercial RWH impacts using slope zone curves. For example, a hypothetical threshold at 50% water demand may be as efficient, with a relatively small variation in slope beyond the threshold. A dramatic increase in impacts was found below 50% of water demand due to input flow inefficiency.
- A threshold can also be devised for an auxiliary commercial RWH system operation. A 50% auxiliary commercial RWH system outperformed MWS in all LCIA categories except Ozone Depletion. Different thresholds determined from the regression equations can be set for different energy intensities.
- Regulatory requirements on water quality, water withdrawal and plumbing codes can also influence impacts due to component size, material, and treatment standards and should be considered when designing a commercial RWH system. When implemented at the watershed-scale, the potential impacts of commercial RWH on freshwater balance, water quality, runoff and sewer overflow, groundwater level (and economic viability) need further attention. Human health (cancer) and ecotoxicity impacts in addition to the cradle-to-cradle analysis are additional research needs.

## Supplementary Material

Supp

## Figures and Tables

**Table 1:** Description of the major components of benchmark commercial rainwater harvesting system and life cycle inventory.

Main component	Sub-component	Material (unit)	Amount	Lifetime (y)	LCI data source
Vortex Filter	Housing	polypropylene (kg)	47.7	40	Ecoinvent (2012)
	Lid	aluminum (kg)	3.6	40	Ecoinvent (2012)
	Intermediate ring	stainless steel (kg)	7.3	40	Ecoinvent (2012)
	Filter insert	stainless steel (kg)	4.1	40	Ecoinvent (2012)
Smoothing inlet	Smoothing inlet	stainless steel (kg)	3.2	40	Ecoinvent (2012)
Floating filter	Filter assembly	stainless steel (kg)	0.7	15	Ecoinvent (2012)
	Hose	food grade reinforced plastic hose (kg)	2.3	15	Ecoinvent (2012)
	Floating ball	polyethylene (kg)	0.2	15	Ecoinvent (2012)
Main pump, 1 hp	Main pump	primarily stainless steel (kg)	18.0	15	Ghimire et al. (2014)
Booster pump, 1 hp	A booster pump	primarily stainless steel (kg)	18.0	15	Ghimire et al. (2014)
Level switch	Float switch and cable	polypropylene (Housing) (kg)	0.9	12.5	Ecoinvent (2012)
Pressure tank	Tank	rolled steel (16 gauge), butyl rubber, copolymer polypropylene (kg)	16.4	50	Ecoinvent (2012)
Bag filter	Bag filter	polypropylene (kg)	0.2	15	Ecoinvent (2012)
	Filter housing	polypropylene (kg)	4.6	15	Ecoinvent (2012)
Ultraviolet (UV) light chamber	Housing	316L stainless steel (kg)	14.6	11	Ecoinvent (2012)
	Bulbs	quartz (kg)	0.9	11	Ecoinvent (2012)
	Quartz sleeves	fused silica (kg)	0.5	11	Ecoinvent (2012)
Solenoid Valve	Valve	brass (kg)	0.5	7.5	Ecoinvent (2012)
Day Tank	high-density polyethylene (HDPE)	PE pipe equivalent length 181 m (kg)	43.2	50	NIST (2013)
Ultrasonic level transmitter	Housing (LU20 Model)	polypropylene (kg)	0.9	15	Ecoinvent (2012)
(sensor) Pipe, collection	2 in Polyvinyl chloride (PVC)	water supply 2 in 1 m - PVC cradle-to-gate (m)	61.0	50	NIST (2013)
Pipe, supply	1.5 in chlorinated PVC	HCWD 1.5 in 1 m- CPVC cradle-to-gate (m)	152.0	50	NIST (2013)
Storage Tank	Fiberglass (FG) Storage Tank	glass fibre (kg)	2773.0	50	NIST (2013)
	Two FG Access Riser (36 in diameter, 3 ft tall)	glass fibre (kg)	113.6	50	Ecoinvent (2012)
	Two FG Access Collars (36 in)	glass fibre (kg)	113.6	50	Ecoinvent (2012)
	Two overflow pipe (8 in 2 ft)	water supply 8 in 1 m - PE cradle-to-gate (kg)	2.3	50	NIST (2013)
Energy usage	Pumping energy	electricity, at residential user (kWh/m^3^)	0.19	N/A	Cashman et al. (2014)

**Table 2:** Sensitivity analysis scenarios.

Commercial RWH system	MWS (Washington D.C. water) system
Storage tank material (Polyethylene and Fiberglass)	–
Commercial RWH energy intensities (0.19–5.51 kWh/m^3^)	MWS energy intensities (0.35–10.15 kWh/m^3^)
Storage tank volume (from 0.25 to 4.5 × benchmark volume)	–
Commercial RWH demand (10%–100% of benchmark demand)	–
Commercial RWH system water loss (0%–30%)	DC Water system water loss (0%–30%)
System service life (50 years versus 75 years)	System service life (100 years)
Auxiliary commercial RWH system (0%–90% MWS)	–

**Table 3:** Life Cycle Impact Assessment (LCIA) tradeoff equations with respect to energy intensities (X, kWh/m^3^). X_c_ and X_m_ are commercial rainwater harvesting (RWH) and municipal water supply (MWS) system energy intensities; Y = LCIA impact score; MAPE = Mean Absolute Percentage Error.

LCIA impact category	Tradeoff condition	Commercial RWH LCIA tradeoff equation	MAPE (%) (commercial RWH equation)	MWS LCIA tradeoff equation	MAPE (%) (MWS equation)
Acidification	X_c_ + *0.05* ≤ X_m_	Y = 4.1 × 10^−3^	0.60	Y = 3.8 × 10^−3^ X + 1.6 × 10^−3^	0.82
		X + 8.4 × 10^−4^			
Energy Demand	Xc + *0.09 ≤ Xm*	Y = 2.0 × 10^1^	0.52	Y = 1.8 × 10^1^ X + 1.9 × 10^0^	2.20
		X + 3.0 × 10^0^			
Eutrophication	*Xc* ≤ *Xm*	Y = 7.8 × 10^−5^	0.35	Y = 7.3 × 10^−5^ X + 1.9 × 10^−4^	1.10
		X + 2.8 × 10^−5^			
Fossil Depletion	*X*_*c*_ *+* 0.07 ≤ X_m_	Y = 3.5 × 10^−1^	0.45	Y = 3.3 × 10^−1^ X + 3.5 × 10^−2^	1.22
		X + 5.6 × 10^−2^			
Freshwater Withdrawal	Regardless of X	Y = 2.4 × 10^−1^	0.32	Y = 2.2 × 10^−1^ X + 1.8 × 10^0^	2.15
		X + 5.9 × 10^−1^			
Global Warming	X_c_ + 0.05 < X_m_	Y = 9.0 × 10^−1^	0.48	Y = 8.4 × 10^−1^ X + 1.1 × 10^−1^	0.38
		X + 1.5 × 10^−1^			
Human Health Criteria	*Xc* ≤ *Xm*	Y = 3.4 × 10^−4^	0.38	Y = 3.2 × 10^−4^X + 1.7 × 10^−4^	0.66
		X + 8.2 × 10^−5^			
Metal Depletion	Regardless of X	Y = 6.6 × 10^−3^	0.15	Y = 6.1 × 10^−3^X + 1.4 × 10^−1^	2.40
		X + 4.2 × 10^−2^			
Ozone Depletion	X_c_ + 0.86 ≤ X_m_	Y = 2.5 × 10^−8^	1.20	Y = 2.4 × 10^−8^ X + 1.7 × 10^−8^	1.38
		X + 3.6 × 10^−8^			
Smog	Xc + 0.05 ≤ Xm	Y = 3.9 × 10^−2^	0.52	Y = 3.6 × 10^−2^ X + 8.3 × 10^−3^	0.12
		X + 1.0 × 10^−2^			
Evaporative Water Consumption	X_c_ + 0.05 ≤ X_m_	Y = 2.7 × 10^−3^	0.26	Y = 2.5 × 10^−3^ X + 2.6 × 10^−6^	0.13
		X + 4.3 × 10^−6^			

**Table 4:** Life Cycle Impact Assessment (LCIA) tradeoff scores of auxiliary commercial rainwater harvesting (RWH) system, augmented with municipal water supply (MWS) from 10% to 90%. *I*_*c*_ and *I*_*m*_ refer to the LCIA impact score per cubic meter water supply of benchmark commercial RWH and MWS systems; % differences are reported for a 50% auxiliary commercial RWH system with respect to *I*_*c*_ given by: [(*I*_*c*_ —Impact value @ 0.5)/*I*_*c*_] × 100.

Impact category	Unit	Benchmark LCIA values		MWS fraction and LCIA scores of the auxiliary commercial RWH system									% Difference @ 50% MWS
		MWS (Im)	Commercial RWH (Ic)	0.1	0.2	0.3	0.4	0.5	0.6	0.7	0.8	0.9	
Acidification	kg SO2 eq	3.0E-03	1.6E-03	1.8E-03	1.9E-03	2.0E-03	2.2E-03	2.3E-03	2.4E-03	2.6E-03	2.7E-03	2.8E-03	−41
Energy Demand	MJ	8.4E+00	6.8E+00	7.0E+00	7.1E+00	7.3E+00	7.4E+00	7.6E+00	7.7E+00	7.9E+00	8.1E+00	8.2E+00	−11
Eutrophication	kg N eq	2.1E-04	4.3E-05	6.0E-05	7.7E-05	9.4E-05	1.1E-04	1.3E-04	1.4E-04	1.6E-04	1.8E-04	2.0E-04	−197
Fossil Depletion	kg oil eq	1.5E-01	1.2E-01	1.3E-01	1.3E-01	1.3E-01	1.3E-01	1.4E-01	1.4E-01	1.4E-01	1.4E-01	1.5E-01	−11
Freshwater Withdrawal	m^3^	1.9E+00	6.4E-01	7.6E-01	8.9E-01	1.0E+00	1.2E+00	1.3E+00	1.4E+00	1.5E+00	1.7E+00	1.8E+00	−101
Global Warming	kg CO2 eq	4.0E-01	3.3E-01	3.3E-01	3.4E-01	3.5E-01	3.6E-01	3.6E-01	3.7E-01	3.8E-01	3.9E-01	4.0E-01	−12
Human Health Criteria	kg PM2.5 eq	2.9E-04	1.5E-04	1.6E-04	1.7E-04	1.9E-04	2.0E-04	2.2E-04	2.3E-04	2.4E-04	2.6E-04	2.7E-04	−47
Metal Depletion	kg Fe eq	1.4E-01	4.3E-02	5.3E-02	6.2E-02	7.2E-02	8.1E-02	9.1E-02	1.0E-01	1.1E-01	1.2E-01	1.3E-01	−109
Ozone Depletion	kg CFC11 eq	2.5E-08	4.1E-08	3.9E-08	3.8E-08	3.6E-08	3.5E-08	3.3E-08	3.2E-08	3.0E-08	2.8E-08	2.7E-08	19
Smog	kg O3 eq	2.1E-02	1.7E-02	1.8E-02	1.8E-02	1.8E-02	1.9E-02	1.9E-02	1.9E-02	2.0E-02	2.0E-02	2.1E-02	−10
Evaporative Water Consumption	m^3^ H2O eq	8.8E-04	5.2E-04	5.5E-04	5.9E-04	6.2E-04	6.6E-04	7.0E-04	7.3E-04	7.7E-04	8.0E-04	8.4E-04	−35
