# Supplementary material for: Life cycle assessment of a commercial rainwater harvesting system compared with a municipal water supply system
Source: J Clean Prod. Author manuscript; Available in PMC 2018 Aug 22. (PMC6104840; doi:10.1016/j.jclepro.2017.02.025)
Supplement: Supp [file NIHMS983356-supplement-Supp.docx]

**SUPPLEMENTARY MATERIAL**

**Title:** **Life Cycle Assessment of a Commercial Rainwater Harvesting System Compared with a Municipal Water Supply System**

*Santosh R. Ghimire ^1^, John M. Johnston^2*^, Wesley W. Ingwersen^3^, and Sarah Sojka^4^*

^1^ORISE Postdoctoral Research Participant, U.S. Environmental Protection Agency, Office of Research and Development, 960 College Station Rd., Athens, GA 30605, USA

E-Mail: sghimire02@gmail.com

^2^U.S. Environmental Protection Agency, Office of Research and Development, 960 College Station Rd., Athens, GA 30605, USA

*Corresponding E-Mail: Johnston.JohnM@epa.gov

^3^U.S. Environmental Protection Agency, Office of Research and Development, 26 W. Martin Luther King Dr., Cincinnati, OH 45268, USA

E-Mail: Ingwersen.Wesley@epa.gov

^4^Randolph College, 2500 Rivermont Ave., Lynchburg, VA 24503, USA

E-Mail: ssojka@randolphcollege.edu

Number of pages: 31

Number of Figures: 16

Number of Tables: 4

Supplementary Material 1 (SM 1) describes the life cycle assessment system boundary, calculation of pumping energy, and sizing of the commercial rainwater harvesting storage tank. SM 2 provides additional details on the life cycle inventory and life cycle assessment of the benchmark commercial rainwater harvesting and municipal water supply systems. SM 3 includes additional information on the benchmark system component performance analyses. SM 4 provides additional information on sensitivity analyses.

**SM 1: Benchmark Systems Definition and Assumptions**

Storage

Treatment

**1 m^3^**

Point-of-use

Distribution

Source water acquisition

**Sensitivity Analysis**

Component disposal

Component transport to user

**Fig. S1.** Life Cycle Assessment system boundary of commercial rainwater harvesting (RWH) and municipal water supply (MWS) systems. Source water for commercial RWH and MWS systems is provided by surface water and rainwater. Figure adapted with permission from (Ghimire et al., 2014). Copyright (2014) American Chemical Society.

***Benchmark commercial rainwater harvesting (RWH) system pumping energy estimation***

Pumping energy intensity (i.e., energy use per unit volume of water, kWh/m^3^), $E_{crwh}$ of commercial RWH system, was expressed as:

$E_{crwh}=\left( \frac{P_{crwh}}{Q_{h}} \right)$ (S1)

where

$E_{crwh}$ = Pumping energy intensity per cubic meter water supply (kWh/m^3^)

$P_{crwh}=$Power (kW) consumed by the pump, estimated using power equation, which also incorporated safety factor of output power,$\alpha$, consistent with Cheng (2002):

$P_{crwh}=\left( \frac{Q x \gamma x H}{1000 x {\eta_{1}x \eta}_{2}} \right)\left( 1+\alpha\right)$ (S2)

where

*Q* = capacity of the pump (1.18 x 10^-04^ m^3^/s)

Pump capacity, *Q,* was estimated by the average daily demand for flushing toilets and urinals for 1,000 employees (500 male and 500 female) in a typical four-story U.S. commercial building, using high-efficiency urinal demand at 0.47 liter/flush (l/f) or 0.125 gallon/flush (g/f) and high-efficiency toilet demand at 4.8 l/f or 1.28 g/f (AWE, 2016). These demands were lower than the EPA WaterSense® maximum average flush rate criteria at 1.9 l/f or 0.5 g/f for water efficiency urinal fixture (USEPA, 2009). Each male employee used 2 urinals and 1 toilet and female employees used 3 toilets every work day, resulting in water flow rate of 1.18 x 10^-04^ m^3^/s [i.e., 500 x (2 x 0.125+1.28 x 1) +500 x (3 x 1.28) = 2,685 gallons/day =1.18 x 10^-04^ m^3^/s]. The system operated 260 days per year (5 days per week x 52 weeks per year).

γ = specific weight of water (9,807 N/m^3^)

H = total pump head (38.2 m) = static head (8.1 m + 0.254 m) + pressure head (21.12 m) + friction losses; with friction losses at 30% of static head and pressure head (i.e., 0.3 x 29.48 m = 8.8 m) (Fig. S2)

η_1_ = pump efficiency (0.65)

η_2_ = Mechanical transmission efficiency (1.00)

α = safety factor of output power (0.2)

The parameters η_1_, η_2_, α, and friction losses were consistent with Cheng (2002).

The estimated pumping energy per m^3^ commercial RWH supply was 0.19 kWh/m^3^.

1.18 x 10^-04^ m^3^/s

**Fig. S2.** Commercial rainwater harvesting system configuration depicting the major parameters used for pumping energy estimation (not to scale).

Commercial RWH energy intensity varies by water demand and system design. In an Australian study, for example, empirical pumping energy ranged from 0.9 kWh/m^3^ for household non-potable use to 4.9 kWh/m^3^ for potable use (Retamal et al., 2009). Others have considered theoretical pumping energy at 0.44 kWh/m^3^ for a three-story office building in U.S. cities (Wang and Zimmerman, 2015) and 0.54 kWh/m^3^ for an office building in the UK (Ward et al., 2012). A literature review by Vieira et al. (2014) reported median theoretical energy intensity at 0.20 kWh/m^3^, which was much lower than other empirical values.

***Benchmark MWS system energy intensity estimation***

We estimated the benchmark MWS energy intensity at 0.35 kWh/m^3^ by combining two energy use sources: Washington Aqueduct energy use (0.20 kWh/m^3^) (Equation S3) and DC Water services energy use (0.15 kWh/m^3^) (Equation S4). The Washington Aqueduct energy intensity,$E_{DC1}$ (0.20 kWh/m^3^), was estimated using volume of water sold to customers and actual energy costs in FY 2012:

$E_{DC1}= \frac{C_{e}}{{V x R}_{e}}$ (S3)

where

$E_{DC1}$*=* Washington Aqueduct energy intensity (kWh/m^3^)

*C_e_* = actual Washington Aqueduct energy costs in FY 2012 ($/y) (obtained from Washington Aqueduct) (WA, 2012)

*V* = volume of water sold to customers in FY 2012: 192,871,598.3 m^3^/y or 50,951.3 MG/y obtained from Washington Aqueduct) (WA, 2012)

*R_e_* = District of Columbia commercial electric rate in 2012 ($0.1202/kWh), obtained from U.S. Energy Information Administration or EIA’s 2012 average monthly commercial bill (USEIA, 2016)

DC Water services energy use intensity,$E_{DC2}$ (0.15 kWh/m^3^), was estimated using available energy use data from the District Department of the Environment (DDOE, 2010) and DC water supply volume in 2012:

$E_{DC2}= \frac{E_{e}}{V}$ (S4)

where

*E_e_* = DC Water services energy use in 2006 (20,534,096 kWh, obtained from District Department of the Environment) (DDOE, 2010)

*V* = DC Water supply in 2012 (139,796,630 m^3^), obtained from Washington Aqueduct (WA, 2012)

We note that the MWS system’s energy intensity varies with water sources and pumping, treatment processes, and storage options, which may be higher if the embedded energy is considered; for example, 4.9 kWh/m^3^ for medium water utility in southern California with 83% imported water (NAP, 2015). Estimated pumping electric energy use by the U.S. public water supply industry, community water systems in 2011 ranged from 0.42 kWh/m^3^ surface water to 0.55 kWh/m^3^ groundwater to 3.17 kWh/m^3^ desalination water, as reported by Electric Power Research Institute and Water Research Foundation (Pabi et al., 2013).

***Benchmark commercial RWH storage tank sizing***

Rainwater Management Solutions provided a design for commercial RWH storage tank (76 m^3^ or 20,000 gallons) using a spreadsheet-based, time series modeling approach (RMS, 2009) that builds on traditional behavioral and mass balance models (Fewkes, 2000; Liaw and Tsai, 2004; Villarreal and Dixon, 2005; Mitchell, 2007; Roebuck and Ashley, 2007). Eighteen scenarios, representing 18 different tank sizes, were modeled using eight-year daily precipitation data, from January 1, 2000 to December 31, 2007, obtained from the National Climatic Data Center for Ronald Reagan National Airport. The average annual precipitation during this period (104 cm) was comparable to the average annual precipitation for the entire period of record (101 cm, 1948-2014). Note that the station was in operation for part of 2015, but a full year of data was not obtained, and 1948 and 1956 were excluded because data was missing for one or more months. The date range chosen to model the RWH system included one unusually dry year (2001, 76 cm of precipitation) and one unusually wet year (2003, 151 cm of precipitation) and therefore represented variability in precipitation with a relatively short period for modeling.

Runoff volume from the roof of a commercial RWH building on day *t* (*RV_t_*, gallons per day) was calculated as:

*RV_t_ = A* x *0.62* x *(P_t_ – 0.01)* x *E* (S5)

where

*A* = roof area in plan view (ft^2^)

*P_t_* = precipitation depth on day *t* (in per day), and

*E* = collection efficiency factor (0.9 was used for this study).

A conversion factor of 0.62 was used to convert inch x ft^2^ to gallons, based on a rule-of-thumb of 0.62 gallons of rainwater collection per square foot of roof area per inch rainfall (TWDB, 2005). Because RWH systems are typically designed so that the “first flush” is diverted from the tank, the first 0.01 in of rainfall on each day was subtracted from the precipitation depth of 0.01 in. This depth is equivalent to 5 gallons per 1,000 ft^2^ of roof area, similar to rules-of-thumb used in designing RWH systems (TWDB, 2005; Boulware et al., 2009).

Overflow volume on day *t* (*O­­_t_*) is calculated using a “spill before supply” approach:

*Ot = Tt-1+RVt – S* (S6)

where

*S* = maximum storage capacity (gallons).

Withdrawals from the system were calculated as a daily demand (*D_t_*) of 2,685 gallons. Water use was calculated assuming 1,000 occupants (five days per week, 8 hours per day), high efficiency fixtures (0.125 g/f urinals and 1.28 g/f toilets), and an equal number of male and female occupants. We assumed 2 urinals and 1 toilet uses per male occupant per day and 3 toilet uses per female occupant per day. The volume of harvested rainwater supplied each day (*RS_t_* in gallons) is calculated as:

*RSt= Dt If Dt ≤ Tt-1 – LW + RVt –Ot* (S7)

*RSt = Tt-1– LW + RVt – Ot If Dt > Tt-1–LW + RVt – Ot* (S8)

These values were used to calculate tank water level as

*Tt = Tt-1 + RVt –Ot –RSt* (S9)

*LW* is a low-water cut-off level (5% of the total tank volume) and *T* is the water level in the tank, both in gallons, and *t* is time in days. This method replicated shut-down of the system when the tank’s water level was low. System performance was evaluated based on volumetric reliability, or the percent of yearly demand supplied by harvested rainwater (rainwater supplied/total demand). Overflow volume per year was the average sum of *O_t_* per year (gallons). A counter identified each day the modeled system overflows and each day the modeled system was not able to meet 100% of demand (“dry” days). Average counts of these days per year were presented as “Overflow days (per year)” and “Dry days (per year),” respectively.

The tank size selected for the LCA was 76 m^3^ (20,000 gallons) based on the modeling approach detailed above (Fig. S3). Annually, this tank size would supply an average of 77% of the total estimated demand: 0.77 x 2,653 m^3^y^-1^ = 2,042.81 m^3^y^-1^ of harvested rainwater. Additional water demand for flushing toilets and urinals would be supplied by the municipal system. Larger tank sizes can meet more of the demand with less runoff but also require more materials, excavation and higher costs for construction. We assumed that the MWS system provided enough pressure head for a building necessary for the additional water demand, thus no extra pumps required.

**Fig. S3.** Percentage demand met by a storage tank in a rainwater harvesting system (adapted with permission from RMS, 2009).

**SM 2: LCA of the Benchmark Systems**


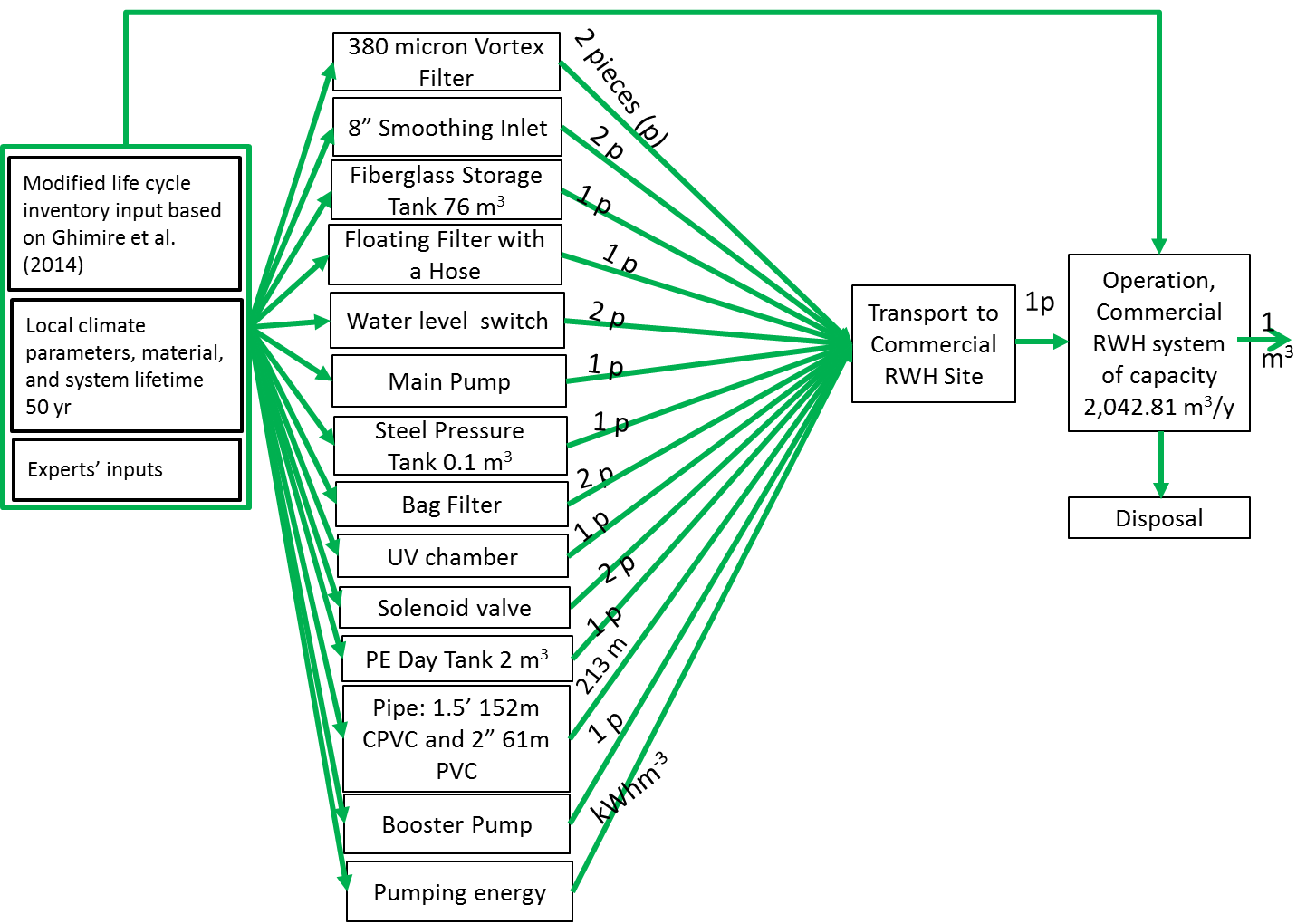


**Fig. S4.** Life Cycle Assessment model of commercial rainwater harvesting system. Figure adapted with permission from (Ghimire et al., 2014). Copyright (2014) American Chemical Society.

**Table S1**

Description of the major components of benchmark municipal water supply system and life cycle inventory (LCI).

| **Main component** | **Sub-component** | **Material (unit)** | **Amount** | **Service life (year)** | **LCI data source** |
| --- | --- | --- | --- | --- | --- |
| Source water acquisition | Potomac River | Water (m^3^) | 1.003 | N/A | N/A |
|  | Source water acquisition infrastructure | Pipes, concrete, brick, excavation, reinforcing steel, Limestone (various units) | 2.401E-07 m/m^3^ pipes to 4.865E-03 kg/m^3^ Limestone | 100 | Cashman et al. (2014) |
| Pump | Acquisition and distribution | primarily stainless steel (pieces) | 24.00 | 15 | Ghimire et al. (2014) |
| Energy usage | Acquisition at water treatment plants and water distribution electricity | electricity, at industrial user (kWh/m^3^) | 0.350 | N/A | DDOE (2010); WA (2012); Cashman et al. (2014); USEIA (2016) |
| Screening and pre-sedimentation | Screening and grit removal infrastructure | Pipes, reinforced concrete, excavation, chromium steel, cast iron, high density polyethylene, aluminum, copper, and steel low alloyed (various units) | 3.116E-08 m/m^3^ pipes to 9.781E-05 kg/m^3^ steel low alloyed | 100 | Cashman et al. (2014) |
|  | Sedimentation infrastructure | Pipes, excavation, brick (various units) | 1.633E-07 m/m^3^ pipes to 3.699E-04 kg/m^3^ brick | 100 | Cashman et al. (2014) |
| Flocculation and sedimentation | Flocculation | Alum coagulant, flocculation infrastructure, polyDADMAC (various units) | 2.907E-07 m/m^3^ pipes to 3.590E-02 lb av/m^3^ Alum | 100 | Cashman et al. (2014) |
|  | Sedimentation | Sedimentation infrastructure (various units) | 1.633E-07 m/m^3^ pipes to 3.699E-04 kg/m^3^ brick | 100 | Cashman et al. (2014) |
| Filtration | Sand | sand, at mine (kg/m^3^) | 6.870E-03 | 100 | Ecoinvent (2012) ; Cashman et al. (2014) |
|  | Filtration infrastructure | Pipes, concrete, excavation, reinforcing steel (various units) | 1.277E-07 m/m^3^ pipes to 7.954E-05 kg/m^3^ reinforcing steel | 100 | Cashman et al. (2014) |
| Primary disinfection | Primary disinfection | Quicklime, lime addition infrastructure, sodium tripolyphosphate, Fluorination, and gaseous chlorine (various units) | 2.705E-03 kg/m^3^ Quicklime to  9.376E-03 lb av/m^3^ hydrogen fluoride | N/A | Cashman et al. (2014) |
| Secondary disinfection | Ammonia | Ammonia, partial oxidation, liquid (kg/m^3^) | 4.762E-04 | N/A | USEPA (1999); Ecoinvent (2012) |
|  | Chlorine | Chlorine, gaseous (kg/m^3^) | 2.000E-03 | N/A | Ecoinvent (2012); DCWSA (2015) |
| Storage tank | Tanks (11 units) | Concrete (m^3^/m^3^) | 3.201E-08 | 100 | DCWSA (2008); Ross (2013) ; Cashman et al. (2014) |
|  |  | Steel (kg/m^3^) | 6.442E-05 | 100 | DCWSA (2008); Ross (2013); Cashman et al. (2014) |
|  |  | Excavation (m^3^/m^3^) | 1.604E-05 | 100 | DCWSA (2008) ; Ross (2013); Cashman et al. (2014) |
| Pipe network | Cast iron (1,772 km) | Cast iron (km/m^3^) | 1.282E-07 | 100 | DCWSA (2008, 2013); Ecoinvent (2012); NIST (2013) |
|  | Ductile iron (211.3 km) | Ductile iron (km/m^3^) | 1.528E-08 | 100 | DCWSA (2008); Cashman et al. (2014) |
|  | Steel (46.0 km) | Proxy Ductile Iron (km/m^3^) | 3.329E-09 | 100 | DCWSA (2008); Ecoinvent (2012) |
|  | Reinforced and prestressed concrete (56.5 km) | Reinforced and prestressed concrete (km/ m^3^) | 4.085E-09 | 100 | DCWSA (2008); Ecoinvent (2012) |
|  | Other (6.3 km) | PVC (km/m^3^) | 4.539E-10 | 100 | DCWSA (2008); Ecoinvent (2012) |
| Valves | 36,000 Valves | Steel (kg/m^3^) | 2.203E-03 | 100 | DCWSA (2008); Cashman et al. (2014) |

The life cycle impact assessment (LCIA) scores of the benchmark commercial RWH and MWS systems are provided in Tables S2 and S3.

**Table S2**

Life Cycle Impact Assessment scores of benchmark commercial rainwater harvesting (RWH) system and components. PVC = polyvinyl chloride; CPVC = chlorinated PVC; FG = fiberglass; UV = ultraviolet.

| Component | Life cycle impact categories and units | | | | | | | | | | |
| --- | --- | --- | --- | --- | --- | --- | --- | --- | --- | --- | --- |
|  | Acidification | Energy Demand | Eutrophication | Fossil Depletion | Freshwater Withdrawal | Global Warming | Human Health Criteria | Metal Depletion | Ozone Depletion | Smog | Evapo. Water Consumption |
|  | kg SO2 eq | MJ | kg N eq | kg oil eq | m^3^ | kg CO2 eq | kg PM2.5 eq | kg Fe eq | kg CFC11 eq | kg O3 eq | m^3^ H2O eq |
| Bag filter | 1.2E-05 | 1.1E-01 | 6.6E-07 | 2.4E-03 | 6.8E-03 | 3.5E-03 | 1.0E-06 | 3.1E-05 | 8.4E-10 | 1.6E-04 | 3.5E-11 |
| CPVC pipe | 3.9E-06 | 2.5E-02 | 4.3E-07 | 5.4E-04 | 6.3E-05 | 2.0E-03 | 1.0E-07 | 4.7E-05 | 5.4E-11 | 1.5E-04 | 4.4E-13 |
| Day tank | 2.1E-06 | 2.4E-02 | 1.5E-07 | 5.5E-04 | 1.7E-06 | 8.4E-04 | 4.0E-08 | 3.2E-06 | 5.3E-14 | 9.0E-05 | 4.8E-16 |
| Energy usage | 7.8E-04 | 3.8E+00 | 1.5E-05 | 6.7E-02 | 4.6E-02 | 1.7E-01 | 6.5E-05 | 1.3E-03 | 4.8E-09 | 7.4E-03 | 5.1E-04 |
| FG storage tank | 6.3E-04 | 2.3E+00 | 1.9E-05 | 4.2E-02 | 4.3E-01 | 1.2E-01 | 5.2E-05 | 6.1E-03 | 3.2E-08 | 7.9E-03 | 3.5E-09 |
| Float filter | 1.3E-05 | 6.5E-02 | 2.1E-06 | 1.3E-03 | 5.3E-03 | 3.8E-03 | 1.2E-06 | 1.2E-04 | 3.7E-10 | 1.6E-04 | 8.7E-08 |
| Level sensor | 1.1E-06 | 1.1E-02 | 6.3E-08 | 2.2E-04 | 6.5E-04 | 3.4E-04 | 9.8E-08 | 3.0E-06 | 8.0E-11 | 1.6E-05 | 3.3E-12 |
| Pressure tank | 1.2E-06 | 6.8E-03 | 8.4E-08 | 1.2E-04 | 1.5E-03 | 3.5E-04 | 1.7E-07 | 1.1E-04 | 4.9E-11 | 1.4E-05 | 1.7E-07 |
| Pump | 1.0E-04 | 1.4E-01 | 2.2E-06 | 2.7E-03 | 6.4E-02 | 7.2E-03 | 1.8E-05 | 2.8E-02 | 3.6E-10 | 6.9E-04 | 7.8E-09 |
| PVC pipe | 2.9E-06 | 2.3E-02 | 2.0E-07 | 5.0E-04 | 5.8E-05 | 1.3E-03 | 4.2E-08 | 4.4E-05 | 4.7E-11 | 1.2E-04 | 4.1E-13 |
| Rain water | 0.0E+00 | 0.0E+00 | 0.0E+00 | 0.0E+00 | 0.0E+00 | 0.0E+00 | 0.0E+00 | 0.0E+00 | 0.0E+00 | 0.0E+00 | 0.0E+00 |
| Smoothing inlet | 6.6E-07 | 3.2E-03 | 5.1E-08 | 5.6E-05 | 9.1E-04 | 1.9E-04 | 9.9E-08 | 7.6E-05 | 1.8E-11 | 7.5E-06 | 1.1E-07 |
| Switch | 3.3E-06 | 3.1E-02 | 1.8E-07 | 6.5E-04 | 1.9E-03 | 9.7E-04 | 2.8E-07 | 8.6E-06 | 2.3E-10 | 4.5E-05 | 9.6E-12 |
| UV light | 2.1E-05 | 1.0E-01 | 1.6E-06 | 1.8E-03 | 2.8E-02 | 6.1E-03 | 3.1E-06 | 2.3E-03 | 5.7E-10 | 2.5E-04 | 3.5E-06 |
| Valve | 2.3E-05 | 9.5E-03 | 4.4E-07 | 1.6E-04 | 1.3E-02 | 5.5E-04 | 3.2E-06 | 5.3E-03 | 4.6E-11 | 1.2E-04 | 2.2E-09 |
| Vortex filter | 2.4E-05 | 1.9E-01 | 1.2E-06 | 3.8E-03 | 3.8E-02 | 6.7E-03 | 2.3E-06 | 3.7E-04 | 1.3E-09 | 3.0E-04 | 4.1E-07 |
| RWH system (Total) | 1.6E-03 | 6.8E+00 | 4.3E-05 | 1.2E-01 | 6.4E-01 | 3.3E-01 | 1.5E-04 | 4.3E-02 | 4.1E-08 | 1.7E-02 | 5.2E-04 |

**Table S3**

Life Cycle Impact Assessment scores of benchmark municipal water supply (MWS) system and components.

| Impact category | Life cycle impact categories and units | | | | | | | | | | |
| --- | --- | --- | --- | --- | --- | --- | --- | --- | --- | --- | --- |
|  | Acidification | Energy Demand | Eutrophication | Fossil Depletion | Freshwater Withdrawal | Global Warming | Human Health Criteria | Metal Depletion | Ozone Depletion | Smog | Evapo. Water Consumption |
| Component | kg SO2 eq | MJ | kg N eq | kg oil eq | m^3^ | kg CO2 eq | kg PM2.5 eq | kg Fe eq | kg CFC11 eq | kg O3 eq | m^3^ H2O eq |
| Energy usage | 1.3E-03 | 6.4E+00 | 2.5E-05 | 1.1E-01 | 7.8E-02 | 2.9E-01 | 1.1E-04 | 2.1E-03 | 8.3E-09 | 1.3E-02 | 8.7E-04 |
| Filtration | 1.7E-06 | 6.6E-03 | 6.4E-08 | 1.3E-04 | 1.2E-03 | 5.5E-04 | 2.1E-07 | 1.0E-04 | 2.6E-11 | 3.3E-05 | 3.7E-08 |
| Flocculation sedimentation | 2.6E-04 | 3.2E-01 | 6.5E-06 | 5.4E-03 | 7.7E-02 | 1.6E-02 | 2.3E-05 | 1.2E-03 | 1.5E-09 | 1.2E-03 | 2.4E-07 |
| Pipe network | 4.8E-05 | 1.8E-01 | 2.0E-06 | 3.8E-03 | 1.7E-02 | 9.8E-03 | 6.7E-06 | 3.5E-03 | 6.4E-10 | 9.0E-04 | 1.5E-06 |
| Primary disinfection | 8.4E-04 | 6.3E-01 | 1.7E-04 | 1.1E-02 | 1.7E-01 | 4.3E-02 | 6.0E-05 | 4.7E-03 | 8.4E-09 | 2.7E-03 | 6.3E-07 |
| Pump | 4.3E-04 | 6.0E-01 | 9.4E-06 | 1.1E-02 | 2.7E-01 | 3.0E-02 | 7.5E-05 | 1.2E-01 | 1.5E-09 | 2.9E-03 | 3.3E-08 |
| Screening and pre-sedimentation | 2.9E-06 | 1.3E-02 | 1.1E-07 | 2.8E-04 | 2.2E-03 | 7.6E-04 | 3.9E-07 | 3.9E-04 | 4.8E-11 | 5.9E-05 | 8.5E-08 |
| Secondary disinfection | 2.2E-05 | 7.7E-02 | 6.3E-07 | 1.3E-03 | 1.8E-02 | 4.1E-03 | 2.7E-06 | 2.5E-04 | 4.6E-09 | 1.5E-04 | 1.4E-10 |
| Source water acquisition | 3.3E-06 | 1.5E-02 | 1.2E-07 | 3.0E-04 | 1.2E+00 | 1.0E-03 | 2.5E-07 | 7.7E-05 | 7.2E-11 | 6.3E-05 | 1.3E-07 |
| Storage tank | 6.7E-07 | 2.4E-03 | 2.2E-08 | 4.9E-05 | 1.1E-03 | 1.6E-04 | 1.8E-07 | 2.5E-04 | 9.5E-12 | 1.1E-05 | 2.2E-11 |
| Valves | 1.9E-05 | 7.5E-02 | 5.5E-07 | 1.5E-03 | 3.5E-02 | 4.7E-03 | 6.2E-06 | 8.4E-03 | 2.5E-10 | 2.6E-04 | 7.5E-10 |
| MWS system (Total) | 3.0E-03 | 8.4E+00 | 2.1E-04 | 1.5E-01 | 1.9E+00 | 4.0E-01 | 2.9E-04 | 1.4E-01 | 2.5E-08 | 2.1E-02 | 8.8E-04 |

**SM 3: Performance Analysis of Benchmark Systems Components**

***Release contribution of benchmark commercial RWH storage tank to Ozone Depletion***

A Sankey diagram illustrates percentage contributions of different flows in commercial RWH storage tank (fiberglass) product system to Ozone Depletion impact. Two major flows contributing to the Ozone Depletion impact of storage tank product system were injection moulding (~73.5%) and glass fibre (~26.5%) (Fig. S5).


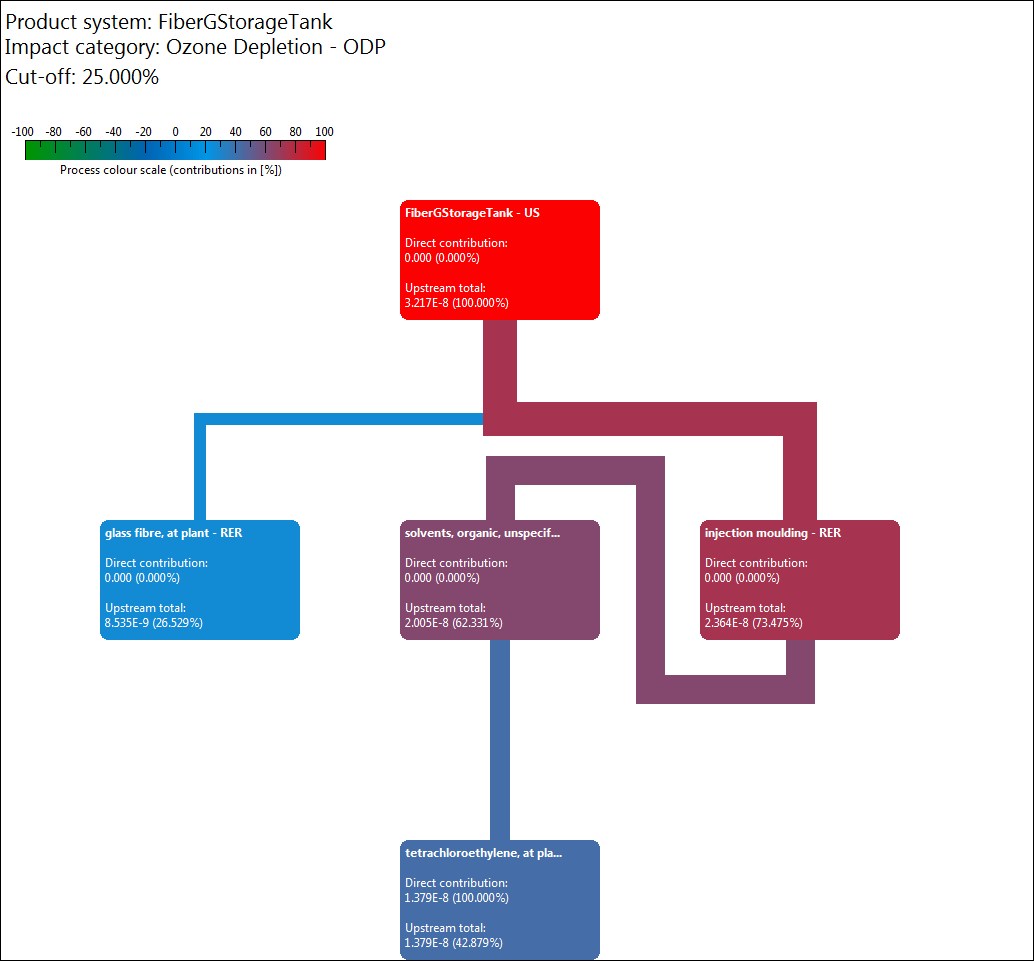


**Fig. S5.** Sankey diagram revealing the percentage contribution of different flows in the benchmark commercial rainwater harvesting storage tank product system’s Ozone Depletion impact category (diagram generated using openLCA with cut-off at 25%) (OpenLCA, 2016).

A closer look at the processes showed that tetrachloroethylene process contributed 42.9% of the storage tank Ozone Depletion impact originating from the injection moulding (Fig. S6), and tetrachloroethylene and dichloromethane (needed for injection moulding of storage tank) contributed to 62.1% of Ozone Depletion (S6).

**Fig. S6**. Major release contributors of benchmark commercial rainwater harvesting fiberglass storage tank to Ozone Depletion impact category. All others include contributing flows lower than 2% individually such as transport (natural gas, pipeline, long distance), crude oil (at production onshore), uranium (enriched 3.0% at enrichment plant), etc. totaling <10%.

***Release contribution of benchmark commercial RWH storage tank to Freshwater Withdrawal***

A closer look at the processes of commercial RWH storage tank showed electricity, hydropower at run-of-river power plant as the major contributing process (93.8%) to Freshwater Withdrawal impact (Fig. S7).

**Fig. S7.** Release contributors to fiberglass storage tank Freshwater Withdrawal impact. All others include contributing flows lower than 1% individually such as electricity and hydropower at pumped storage power plant, etc. totaling <3%.

***Top five process contributors to benchmark commercial RWH energy usage***

A further analysis of commercial RWH energy usage (pumping energy) processes revealed electricity, coal as dominant process for five impact categories: Acidification (74.3%), Eutrophication (49%), Global Warming (67.8%), Human Health Criteria (68.9%), and Smog (55.4%) (Fig. S8).

**Fig. S8.** Top five process contributors to benchmark commercial rainwater harvesting system pumping energy life cycle impact categories.

***Release contribution of benchmark commercial RWH pump to Metal Depletion***

A closer look at the processes of commercial RWH pump to the dominant Metal Depletion impact indicated copper concentrate as the major (68.0%) release contributor (Fig. S9).

**Fig. S9.** Major release contributors of benchmark commercial rainwater harvesting system Pump to Metal Depletion impact category. All others include contributing flows lower than 1% individually, such as Molybdenum concentrate, main product tin, at regional storage, etc. totaling <2%.

**SM 4: Sensitivity Analysis of Benchmark Systems**

***Sensitivity of storage tank materials on the LCIA of commercial RWH system***

**Table S4**

Description of Storage tank materials.

| Main component | Sub-component | Material | Mass (kg) | Source |
| --- | --- | --- | --- | --- |
| Fiberglass (FG) Storage Tank and accessories | FG Storage Tank | Glass fibre | 2,773 | Ecoinvent (2012) |
|  | Two FG Access Riser (36 in Diameter 3 ft tall) | Glass fibre | 113.6 | Ecoinvent (2012) |
|  | Two FG Access Collars (36 in Diameter) | Glass fibre | 113.6 | Ecoinvent (2012) |
|  | Two overflow pipe (8 in Diameter 2 ft high-density polyethylene or HDPE) | Water supply 1 in 1 m - PE cradle-to-gate: equivalent length of 4 x 8 ft x 0.3048 m/ft = 9.75 m | 2.3 | NIST (2013) |
| Polyethylene (PE) Storage Tank and accessories (BEES database) | PE Storage Tank | Water supply 1 in 1 m - PE cradle-to-gate: equivalent length 10,507 m @ 0.16 lb/ft | 2,506 | NIST (2013); Plastic-Mart (2016) |
|  | Two PE Access Riser (36 in Diameter 3 ft tall) | Water supply 1 in 1 m - PE cradle-to-gate: equivalent length of 36 x 2 x 3 ft x 0.3048 m/ft | 15.7 | NIST (2013) |
|  | Two PE Access Collars (36 in Diameter x 3 ft) | Water supply 1 in 1 m - PE cradle-to-gate: equivalent length of 36 x 2 x 3 ft x 0.3048 m/ft | 15.7 | NIST (2013) |
|  | Two overflow pipe (8 in 2 ft HDPE) | Water supply 8 in 1 m - PE cradle-to-gate: equivalent length of 8 x 2 x 2 ft x 0.3048 m/ft | 2.3 | NIST (2013) |
| PE Storage Tank and accessories (Ecoinvent database) | PE Storage Tank | Polyethylene, HDPE, granulate, at plant | 2,506 | Ecoinvent (2012); (Plastic-Mart, 2016) |
|  | Two PE Access Riser (36 in Diameter 3 ft tall) | Polyethylene, HDPE, granulate, at plant | 15.7 | Ecoinvent (2012) |
|  | Two PE Access Collars (36 in Diameter) | Polyethylene, HDPE, granulate, at plant | 15.7 | Ecoinvent (2012) |
|  | Two overflow pipe (8 in 2 ft long HDPE) | Polyethylene, HDPE, granulate, at plant: @ (0.16 lb/ft x 8 x 4 ft x 1kg/2.2 lb) | 2.3 | NIST (2013) |

We performed an LCIA of commercial RWH storage tank (76 m^3^) materials, fiberglass and polyethylene (PE) with Ecoinvent (2012) and the Building for Environmental and Economic Sustainability (BEES) database of the National Institute of Standards and Technology (NIST, 2013) (Table S4).

The LCIA of the storage tanks (fiberglass and PE materials) indicated mixed results: fiberglass dominated in nine impact categories and PE dominated in the remaining two, Energy Demand and Fossil Depletion, regardless of data source (Fig. S10).

**Fig. S10.** Life Cycle Impact Assessment **(**LCIA) comparison of commercial rainwater harvesting storage tank materials fiberglass and polyethylene with two different data sources, the Building for Environmental and Economic Sustainability (BEES) database and Ecoinvent database (EI). LCIA impact scores were normalized by maximum value per category.

***Sensitivity of storage tank volume on the LCIA of commercial RWH system***

We varied the benchmark commercial RWH storage tank volume from 19 m^3^ to 342 m^3^ (0.25 to 4.5 times the benchmark volume of 76 m^3^), keeping system demand and other design parameters unchanged. The maximum volume of 342 m^3^ (i.e., 4.5 x 76 m^3^) equals 100% of water demand met by commercial RWH. The normalized LCIA with respect to maximum impact at 342 m^3^ revealed linear variation in impacts with zones of Mild, Moderate, and High slopes (Fig. S11), similar to energy usage variation zones.

**Fig. S11.** Sensitivity analysis of commercial rainwater harvesting (RWH) storage tank volume variation to Life Cycle Impact Assessment (LCIA) of benchmark commercial RWH system. LCIA impact scores were normalized by maximum value of 342 m^3^.

The storage tank’s Evaporative Water Consumption impact was found to be insensitive to the commercial RWH system because its actual Evaporative Water Consumption (3.46 x 10^-9^ m^3^) was negligible when compared to commercial RWH’s 0.00051 m^3^ (Fig. S12). Further, the Ecoinvent 2.2 database was not comprehensive in its inclusion of evaporative water losses, partially contributing to underestimates of actual Evaporative Water Consumption.

**Fig. S12.** Comparison of Evaporative Water Consumption impact of commercial rainwater harvesting (RWH) system with storage tank and pumping energy.

***Sensitivity of water demand on the LCIA of commercial RWH system***

We varied the benchmark commercial RWH system water demand (2,653 m^3^/y) from 265.3 m^3^/y to 2,653 m^3^/y (10% to 100%). Each commercial RWH component input except pump and pumping energy was influenced by this variation because flow inputs for the components were normalized by water demand. Pump and pumping energy intensity were kept constant, regardless of the demand variation range. Because input flows of each component were normalized by water demand, the impacts also varied inversely (Fig. S13a, b).

50% Threshold

**Fig. S13 a.** Sensitivity analysis of benchmark commercial rainwater harvesting system annual water demand variation (10%-100% of total system demand at 2,653 m^3^/y) to Life Cycle Impact Assessment (LCIA). LCIA impacts were normalized by maximum value at lowest annual water demand of 10% of 2,653 m^3^/y.

Notice that the high variation zone was still Ozone Depletion impact because the storage tank was the dominant component for this category; the minor variation in Evaporative Water Consumption slope occurred for the same reason discussed in the storage tank sensitivity analysis in Fig. S12.

**Fig. S13 b.** Sensitivity analysis of benchmark commercial rainwater harvesting system annual water demand variation with inverse scale of percentage (i.e., from 1/10% to 1/100% of benchmark system demand at 2,653 m^3^/y) to Life Cycle Impact Assessment (LCIA). LCIA impacts were normalized by maximum value at lowest annual water demand of 10% of 2,653 m^3^/y.

***Sensitivity of water losses on the LCIA of commercial RWH and MWS systems***

We varied water losses from 0% to 30% throughout the benchmark commercial RWH and MWS systems. Normalized LCIA results with respect to maximum impact values at 30% water loss in benchmark systems indicated linear variation in all impacts which was expected due to the corresponding linear variation in input flows. Because water loss linearly altered the input of each component, LCIA impacts also varied for both systems (Fig. S14); sensitivity of water loss to Ozone Depletion impact is presented as an example, and all other impact categories followed the same trend (not shown).

**Fig. S14.** Sensitivity analysis of water losses in benchmark commercial rainwater harvesting (CRWH) and municipal water supply (MWS) systems. Ozone Depletion impact normalized with respect to maximum Ozone Depletion impact, with highest water losses at 30%.

***Sensitivity of service life on the LCIA of commercial RWH system***

We performed the sensitivity analysis of benchmark commercial RWH system to service lives of 50 and 75 years. Annual LCIA results were normalized with respect to maximum impact values at shorter service life of 50 years. Analysis showed lower annual LCIA impacts for the commercial RWH system with a higher service life of 75 years ranging from 66.9% (Evaporative Water Consumption) to 99.0% (Metal Depletion) (Fig. S15).

**Fig. S15.** Sensitivity of benchmark commercial rainwater harvesting system to system service life, T. Annual Life Cycle Impact Assessment (LCIA) scores were normalized by maximum LCIA scores at T = 50 year (y).

***Tradeoff analysis of auxiliary commercial RWH system augmented with municipal water supply***

Analysis of the auxiliary commercial RWH system provided additional insights into LCIA tradeoffs. An auxiliary commercial RWH with 50% MWS reduced Ozone Depletion impact by 19% showing increases in all other impacts with respect to benchmark commercial RWH impacts (Fig. S16). The slope of auxiliary commercial RWH system’s Ozone Depletion impact was reversed because the benchmark commercial RWH system’s Ozone Depletion impact was greater than that of the MWS system.

**Fig. S16.** Tradeoff analysis of an auxiliary commercial rainwater harvesting (RWH) system augmented with municipal water supply (MWS). Life Cycle Impact Assessment (LCIA) scores were normalized by maximum LCIA value at 100% MWS.

***LCIA normalization***

For all analyses, the normalized impact was calculated with respect to maximum impact, as given by Equation (S10):

$I_{n}= \frac{I}{I_{max}} x 100$ (S10)

where for a specified LCIA impact category,

*I_n_* = normalized impact (dimensionless, %)

*I_max_* = maximum impact (impact/m^3^)

*I* = current impact (impact/m^3^)

***Mean absolute percentage error (MAPE)***

Mean absolute percentage error (MAPE) was estimated as:

$MAPE= \frac{100}{n} x \sum_{n=1}^{15} \frac{|M-F|}{M}$ (S11)

where

n = Number of variants (15)

M = Modeled LCIA score

F = Forecast LCIA score using the tradeoff equation

**REFERENCES**

AWE, 2016. Office Building Introduction. [www.allianceforwaterefficiency.org/office_buildings.aspx](http://www.allianceforwaterefficiency.org/office_buildings.aspx) (April 13, 2016).

Boulware, E.B., Pope, T., Lye, D., Kniffen, B., Wheeler, J., Morris, W., Jennings, R., Shultz, J., Winters, W., Daily, C., Knight, J., 2009. Rainwater catchment design and installation standards. American Rainwater Catchment Systems Association and American Society of Plumbing Engineers, USA.

Cashman, S., Gaglione, A., Mosley, J., Weiss, L., Hawkins, T.R., Ashbolt, N.J., Cashdollar, J., Xue, X., Ma, C., Arden, S., 2014. Environmental and cost life cycle assessment of disinfection options for municipal drinking water treatment. U.S. Environmental Protection Agency, Cincinnati, OH USA.

Cheng, C.-L., 2002. Study of the inter-relationship between water use and energy conservation for a building. Energy and Buildings 34, 261-266.

DCWSA, 2008. Independent Engineering Inspection of the District Of Columbia Water and Sewer Authority’s Wastewater and Water Systems. District of Columbia Water and Sewer Authority Washington DC.

DCWSA, 2013. Independent Engineering Inspection of DC Water’s Wastewater and Water Systems, findings and recommendations, Final Report. District of Columbia Water and Sewer Authority Washington DC.

DCWSA, 2015. Chlorine, Disinfection Performance. [www.dcwater.com/waterquality/chlorine.cfm](https://www.dcwater.com/waterquality/chlorine.cfm), (Oct. 7, 2015).

DDOE, 2010. District of Columbia Greenhouse Gas Emissions Inventory 2006 Emissions Baseline. District Department of the Environment, Washington, DC.

Ecoinvent, 2012. Ecoinvent Centre. [www.ecoinvent.org](http://www.ecoinvent.org) (July 2, 2012).

Fewkes, A., 2000. Modelling the performance of rainwater collection systems: towards a generalised approach. Urban Water 1, 323-333.

Ghimire, S.R., Johnston, J.M., Ingwersen, W.W., Hawkins, T.R., 2014. Life Cycle Assessment of Domestic and Agricultural Rainwater Harvesting Systems. Environmental Science & Technology 48, 4069-4077.

Liaw, C.H., Tsai, Y.L., 2004. Optimum storage volume of rooftop rain water harvesting systems for domestic use. Wiley Online Library.

Mitchell, V.G., 2007. How important is the selection of computational analysis method to the accuracy of rainwater tank behaviour modelling? Hydrol Process 21, 2850-2861.

NAP, 2015. Using Graywater and Stormwater to Enhance Local Water Supplies: An Assessment of Risks, Costs, and Benefits. The National Academies of Sciences, Engineering, and Medicine, Washington, DC.

NIST, 2013. Building for Environmental and Economic Sustainability (BEES). [www.nist.gov/el/economics/BEESSoftware.cfm](http://www.nist.gov/el/economics/BEESSoftware.cfm) (Feb 15, 2013).

OpenLCA, 2016. openLCA 1.4.2. www.openlca.org/.

Pabi, S., Amarnath, A., Goldstein, R., Reekie, L., 2013. Electricity Use and Management in the Municipal Water Supply and Wastewater Industries.

Plastic-Mart, 2016. 20000 Gallon Vertical Plastic Storage Tank. [www.plastic-mart.com/product/6470/20000-gallon-vertical-plastic-storage-tank-43825](http://www.plastic-mart.com/product/6470/20000-gallon-vertical-plastic-storage-tank-43825) (Aug. 2, 2016).

Retamal, M., Glassmire, J., Abeysuriya, K., Turner, A., White, S., 2009. The Water-Energy Nexus: Investigation into the Energy Implications of Household Rainwater Systems. Institute for Sustainable Futures, University of Technology, Sydney.

RMS, 2009. Tank sizing spreadsheet. Rainwater Management Solutions, Salem, VA.

Roebuck, R., Ashley, R., 2007. Predicting the hydraulic and life-cycle cost performance of rainwater harvesting systems using a computer based modelling tool. Water Practice and Technology 2, wpt2007046.

Ross, R.B., 2013. Metallic materials specification handbook. Springer Science & Business Media.

TWDB, 2005. The Texas Manual on Rainwater Harvesting, 3rd ed. Texas Water Development Board, Austin, USA.

USEIA, 2016. Electric Sales, Revenue, and Average Price www.eia.gov/electricity/sales_revenue_price/index.cfm (April 13, 2016).

USEPA, 1999. Alternative Disinfectants and Oxidants Guidance Manual. Office of Water, U.S. Environmental Protection Agency, Washington DC.

USEPA, 2009. WaterSense Specification for Flushing Urinals. [www3.epa.gov/watersense/docs/urinal_finalspec508.pdf](https://www3.epa.gov/watersense/docs/urinal_finalspec508.pdf) (August 14, 2009).

Vieira, A.S., Beal, C.D., Ghisi, E., Stewart, R.A., 2014. Energy intensity of rainwater harvesting systems: A review. Renewable and Sustainable Energy Reviews 34, 225-242.

Villarreal, E.L., Dixon, A., 2005. Analysis of a rainwater collection system for domestic water supply in Ringdansen, Norrköping, Sweden. Building and Environment 40, 1174-1184.

WA, 2012. Washington Aqueduct Annual Financial Report Fiscal Year 2012. Finance and Accounting Office, Washington Aqueduct, Washington, DC.

Wang, R., Zimmerman, J.B., 2015. Economic and environmental assessment of office building rainwater harvesting systems in various U.S. cities. Environ Science& Technol 49, 1768-1778.

Ward, S., Butler, D., Memon, F.A., 2012. Benchmarking energy consumption and CO2 emissions from rainwater-harvesting systems: an improved method by proxy. Water and Environment Journal 26, 184-190.
